# Evidence that the pituitary gland connects type 2 diabetes mellitus and schizophrenia based on large-scale trans-ethnic genetic analyses

**DOI:** 10.1186/s12967-022-03704-0

**Published:** 2022-11-03

**Authors:** Lei Cai, Yanlan Sun, Yonglin Liu, Wenzhong Chen, Lin He, Dong-Qing Wei

**Affiliations:** 1 Bio-X Institutes, Key Laboratory for the Genetics of Developmental and Neuropsychiatric Disorders (Ministry of Education), State Key Laboratory of Microbial Metabolism & Joint International Research Laboratory of Metabolic and Developmental Sciences, Collaborative Innovation Center for Genetics and Development, School of Life Sciences and Biotechnology, Shanghai Jiaotong University, 200240 Shanghai, China; 2 State Key Laboratory of Microbial Metabolism & Joint International Research Laboratory of Metabolic and Developmental Sciences, School of Life Sciences and Biotechnology, Shanghai Jiao Tong University, Shanghai, China; 3 Sanya Women and Children’s Hospital managed by Shanghai Children’s Medical Center, 572022 Sanya, Hainan China; 4 Shanghai Mental Health Center, Shanghai Jiaotong University School of medicine, 600 Wanpingnan Road, 200240 Shanghai, China; 5 Shanghai Center for Women and Children’s Health, 200062 Shanghai, China

**Keywords:** GWAS, TWAS, T2DM, Schizophrenia, Pituitary gland

## Abstract

**Background:**

Previous studies on European (EUR) samples have obtained inconsistent results regarding the genetic correlation between type 2 diabetes mellitus (T2DM) and Schizophrenia (SCZ). A large-scale trans-ethnic genetic analysis may provide additional evidence with enhanced power.

**Objective:**

We aimed to explore the genetic basis for both T2DM and SCZ based on large-scale genetic analyses of genome-wide association study (GWAS) data from both East Asian (EAS) and EUR subjects.

**Methods:**

A range of complementary approaches were employed to cross-validate the genetic correlation between T2DM and SCZ at the whole genome, autosomes (linkage disequilibrium score regression, LDSC), loci (Heritability Estimation from Summary Statistics, HESS), and causal variants (MiXeR and Mendelian randomization, MR) levels. Then, genome-wide and transcriptome-wide cross-trait/ethnic meta-analyses were performed separately to explore the effective shared organs, cells and molecular pathways.

**Results:**

A weak genome-wide negative genetic correlation between SCZ and T2DM was found for the EUR (r_g_ = − 0.098, *P* = 0.009) and EAS (r_g_ =- 0.053 and *P* = 0.032) populations, which showed no significant difference between the EUR and EAS populations (*P* = 0.22). After Bonferroni correction, the r_g_ remained significant only in the EUR population. Similar results were obtained from analyses at the levels of autosomes, loci and causal variants. 25 independent variants were firstly identified as being responsible for both SCZ and T2DM. The variants associated with the two disorders were significantly correlated to the gene expression profiles in the brain (*P* = 1.1E-9) and pituitary gland (*P* = 1.9E-6). Then, 61 protein-coding and non-coding genes were identified as effective genes in the pituitary gland (*P* < 9.23E-6) and were enriched in metabolic pathways related to glutathione mediated arsenate detoxification and to D-myo-inositol-trisphosphate.

**Conclusion:**

Here, we show that a negative genetic correlation exists between SCZ and T2DM at the whole genome, autosome, locus and causal variant levels. We identify pituitary gland as a common effective organ for both diseases, in which non-protein-coding effective genes, such as lncRNAs, may be responsible for the negative genetic correlation. This highlights the importance of molecular metabolism and neuroendocrine modulation in the pituitary gland, which may be responsible for the initiation of T2DM in SCZ patients.

**Supplementary Information:**

The online version contains supplementary material available at 10.1186/s12967-022-03704-0.

## Background

As human economic development as progressed, both schizophrenia (SCZ) and type 2 diabetes mellitus (T2DM), complex polygenic inherited disorders, have become growing challenges that, to date, lack effective solutions [1, 2]. Accumulating evidence from clinical samples demonstrates that the prevalence of T2DM in patients with SCZ is elevated 2 to 3 times compared with the general population, whereas the aetiology for the co-occurrence of SCZ and T2DM is multifactorial [3]. Recent studies have shown that drug-naive patients with their first episode of SCZ have an increased risk of T2DM [4, 5]. Moreover, the increased risk of T2DM is more apparent in young adults with SCZ [3, 6]. Therefore, a better understanding of the genetic relationship between and common genetic basis of SCZ and T2DM is pivotal for providing insights into the treatment and prevention of these diseases.

Since inherited factors rarely correlate with confounders and exhibit no reverse causation, several studies with limited sample sizes have investigated the involved genes common to both SCZ and T2DM and have reported negligible genetic correlations between SCZ and T2DM [7, 8]. This conflicts with a weak genome-wide negative correlation between SCZ and T2DM (rg = − 0.07 and P = 0.002) identified in a forthcoming article with a large-scale sample size of European (EUR) subjects[9]. These inconsistent genetic analysis results may be because the use of limited sample sizes and certain analytical methods potentially result in underpowered correlation analyses, produce bias, and overestimate the results. Moreover, genome-wide association studies (GWASs) involving different population groups can provide samples from global populations to address some of the existing Eurocentric bias, which enhances the ability to identify disease associations and ensures that the findings are mostly relevant to all populations [10]. Thus, a large-scale trans-ethnic genetic analysis can provide new and cross-validated evidence by employing a range of complementary approaches.

In this study, based on GWAS summary data from European (EUR) and East Asian (EAS) populations including a total of 1,466,906 subjects, multiple complementary genomic analysis approaches were utilized to explore the genetic basis for T2DM and SCZ at different levels, such as the whole-genome, autosomes, loci and causal variants. We aimed to provide more evidence of the genetic basis for the comorbidity of these two diseases. First, in addition to performing a linkage disequilibrium (LD) score regression analysis (LDSC) to estimate the genome-wide correlation of SCZ with T2DM, a stratified autosome-based LDSC was used to estimate autosome correlation. Second, Heritability Estimation from Summary Statistics (HESS) method was performed to estimate the locus-level genetic correlation. Third, based on the causal variants of each disease, polygenic overlap and Mendelian randomization (MR) analyses were performed to examine the genetic link between these two diseases. Furthermore, to identify the basic mechanisms underlying the comorbidity of SCZ and T2DM, a genome-wide cross-trait/ethnic meta-analysis was performed to identify the pleiotropic genes shared between SCZ and T2DM and to determine the common effective organs and blood cell types. Finally, a cross-trait/ethnic meta-analysis based on transcriptome-wide association study (TWAS) data was carried out to explore the canonical pathways in the effective organs (Figure S1).

## Data and methods

### GWAS data sets for SCZ and T2DM

GWAS data were collected from the databases of the Psychiatric Genomics Consortium (PGC) and the DIAbetes Genetics Replication And Meta-analysis (DIAGRAM) consortium upon request. The EAS GWAS T2DM dataset included 433, 540 subjects from 23 projects, and the EUR T2DM dataset contained 898, 130 subjects from 32 projects. The EAS GWAS SCZ dataset included 58, 140 subjects, and the EUR SCZ dataset contained 77, 096 subjects[10–13]. The detailed demographic characteristics and quality controls are summarized in Supplementary Material part 1.1.

The quality of the GWAS datasets was controlled by applying the following data filters: variants with INFO ≥ 0.80 if they existed were filtered in; variants with consistent alleles among each dataset were checked to adjust two situations: palindromic alleles and opposite alleles. In total, 8, 335, 938 variants for SCZ_EAS and 9, 745, 488 for SCZ_EUR, 11, 825, 585 for T2DM_EAS and 13, 583, 104 for T2DM_EUR were considered for the next analysis.

### Genetic correlation analysis

First, the heritability of each disorder (single-trait) and the genome-wide correlation (r_g_) between SCZ and T2DM in either the EAS or EUR samples were estimated using linkage disequilibrium (LD) score regression software (LDSC, v1.0.1) and the precomputed LD scores for each population as a reference, which were obtained from the 1000 Genomes (1kG) project phase 3 [7, 14]. Prior to analysis, we filtered out those SNPs that were within the major histocompatibility complex (MHC) but were not within HapMap3 or had a MAF < 5% within the 1kG EUR or EAS reference samples. Furthermore, Fisher’s Z score transformed from r_g_ was calculated to compare the significance of the difference in the genetic correlations between the EAS and EUR samples (Supplementary materials part 1.2). Moreover, partitioned LDSC analysis was performed to estimate the genetic correlation of these two diseases for each autosome.

Second, the Heritability Estimation from Summary Statistics software package (HESS, v0.5.3-beta) was applied to explore the local-level heritability of each disorder and the genetic correlation between SCZ and T2DM within independent LD blocks obtained from the 1kG reference panel in three steps: S1, preparing the LD block and eigenvalues; S2, estimating the local SNP-heritability of each trait; and S3, estimating the local genetic covariance and standard error [15]. A total of 1, 443 and 1, 702 approximately independent LD blocks for the EAS and EUR samples, respectively, were checked as genome partition loci by HESS [16]. The local genetic correlation was calculated with the following formula:

1 $${r}_{L}=\frac{{cov}_{L}}{\sqrt{{{h}_{L}^{2}\left(SCZ\right){ h}_{L}^{2} \left(T2D\right)}_{ }}}$$

Here, cov_L_ is the local genetic covariance obtained from the third step of HESS, and ${\text{h}}_{L}^{2}$(SCZ) and ${\text{h}}_{L}^{2}$(T2DM) are the estimated local heritability of each disease obtained from the second step.

Finally, to qualify the polygenic overlap of these two disease, the total number of shared and trait-specific causal variants between the two diseases was estimated using MiXeR v1.3 with default parameters [17]. To avoid taking infinitesimally small effects, the presented numbers of causal variants accounted for more than 22.6% of their total estimate and jointly accounted for 90% of the heritability of the SNP in each disease.

### Mendelian randomization analysis

To obtain reliable and noteworthy results, bidirectional MR analyses were performed with multiple MR methods based on different assumptions about horizontal pleiotropy. First, GCTA v1.93.3beta2 software was used to analyze the bidirectional causal links between SCZ and T2DM with the generalized summary-data-based Mendelian randomization (GSMR) method [18] with the following parameters: *P* ≤ 5 × 10^− 8^ as the GWAS threshold to select variants for clump analysis; *r*^2^ ≤ 0.05 as the LD threshold to identify independent SNPs based on the 1kG Project (phase 3) population reference; *P* = 0.01 as the threshold for heterogeneity in dependent instruments (HEIDI) outlier analysis to remove horizontal pleiotropic SNPs; and 10 as the minimum number of significant and independent instrumental SNPs required for the MR analysis. Then, three more methods, i.e., inverse variance weighting (IVW), maximum likelihood (ML), and weighted median (WMe), were utilized to explore putative causal relationships between SCZ and T2DM in the EUR population using the R package TwoSampleMR with the following parameters: *P* ≤ 5 × 10^− 8^ and *r*^2^ ≤ 0.05 [19]. MR-Egger regression and MR-PRESSO models with the corresponding R packages were used to determine directional pleiotropy [20].

### Genome-wide cross-trait/ethnic meta-analysis

The Cross Phenotype Association (CPASSOC) method [21] was employed to identify shared variants between SCZ and T2DM. This method allows the presence of heterogeneous effects across traits and provides statistical S_Het_ and P values weighted by sample size. The Z score for each variant for SCZ or T2DM from each population was used as the input source data for the cross-trait/ethnic meta-analysis. A significance level of *P* = 5 × 10^− 8^ was applied as in the GWAS.

Among the genome-wide cross-trait/ethnic significant SNPs, independent cross-trait significant SNPs that met the following two criteria were prioritized: (1) the SNP was not identified as significant in the single-trait GWAS, and (2) the SNP was independent with LD r^2^ < 0.05 within 1,000-kb windows based on the 1kG population reference, evaluated by LD clumping using PLINK v1.970.

### Positional gene mapping

To map and prioritize genes, MAGMA gene analysis was performed with the SNP-wide mean model using the 1kG Phase 3 population reference [22]. During the analysis, genes within 100 kb of each candidate SNP were mapped and prioritized, which were in LD with genome-wide significant SNPs at the adjusted r^2^ threshold using the Functional Mapping and Annotation (FUMA) GWAS web tool [23]. Furthermore, to identify the tissue specificity of the SCZ and T2DM cross-traits, MAGMA gene property analyses in FUMA were performed to test correlations between tissue specific gene expression profiles and trait-gene associations based on the full distribution of SNP *P* values.

### Cell type-specific analysis

To determine the effective cell type in human peripheral blood mononuclear cells (PBMCs) for both SCZ and T2DM, 10x genomics’ single-cell RNA-seq (scRNA-seq) data were extracted [24]. Based on the regression model with SNPs, MAGMA gene-property analysis was performed to test the cell type-specificity of phenotypes with GWAS summary statistics using the FUMA platform [23].

### Transcriptome-wide cross-trait/ethnicity meta-analysis

The Functional Summary-based Imputation (FUSION) package was used to perform TWAS analysis [25]. The pituitary gland-related expression weights were prepared with the aid of the FUSION website and were then integrated with the GWAS data to identify the gene expression associated with either disease in either population.

Then, association analysis was performed on SubSets (ASSET v2.4.0), which can exhaustively explore all possible subsets of inputs to identify the strongest association signal in both positive and negative directions [26]. The above TWAS data and sample size information for SCZ_EAS, T2DM_EAS, SCZ_EUR and T2DM_EUR were input as trait 1 to trait 4, and the two-sided statistic was generated with the default setting parameters. Finally, we took the beta and *P* values for each gene to use in the subsequent Ingenuity Pathway analysis (IPA).

### IPA analysis

With the above effective genes in the pituitary gland identified for both SCZ and T2DM, IPA software (Ingenuity Systems; Qiagen China Co., Ltd.) was employed to perform the core analysis on the measurement of expression logOR as previously described [27].

### Statistical analyses

All statistical analyses were performed using R 4.1.1 and/or Python 2.7/3.7 in the Linux environment, which was run in the π 2.0 cluster supported by the Center for High Performance Computing at Shanghai Jiao Tong University. Detailed descriptions of the genetic correlation analysis, MR analysis and GWCTM are provided in the Supplementary Materials. *P* values < 0.05 were considered statistically significant, and multiple tests were adjusted by the Bonferroni method to reduce the risk of type I statistical error.

## Results

### Genetic correlations between SCZ and T2DM

The results of the single-trait LDSC showed that the genome-wide SNP heritability was 44.22 ± 2.33% and 45.06 ± 1.69% for SCZ, and 7.98 ± 0.49% and 4.45 ± 0.27% for T2DM, in the EAS and EUR samples, respectively. The intercepts of the LD score regression were ≤ 1.003 and 1.05 separately in the EAS and EUR samples, indicating slight bias from population stratification and cryptic relatedness[28]. A negative genetic correlation between SCZ and T2DM was found (r_g_ = − 0.053 and − 0.098, *P* = 0.032 and 0.009, for the EAS and EUR samples, respectively, **Table **1), with no significant difference between the two populations based on Fisher’s Z-transformation method (Z score = 1.23, *P* = 0.22)[29]. Only in the EUR samples did the negative genetic correlation of SCZ with T2DM remain Bonferroni significant (*P* < 0.05/2 = 0.025). The intercept of genetic covariance between SCZ and T2DM for each population was ≤ 0.01, indicating negligible sample overlap between these two diseases in the current analysis[7].

**Table 1:** Genetic correlation and polygenic overlap analyses of the SCZ and T2D

Traits	Genetic correlation analysis			Polygenic overlap analysis				
	**r**_**g**_ **± se**	**P**	**P**_**Bonferroni**_	**N**_**SCZ**_ **±se**	**N**_**T2D**_ **±se**	**N**_**SCZ−T2D**_ **±se**	**ρ**_**SCZ−T2D**_ **±se**	**r**_**g**_ **±se**
SCZvs T2D (EAS)	-0.054 ± 0.025	0.032	0.064	6566.74 ± 401.14	580.35 ± 273.78	665.77 ± 259.24	-0.28 ± 0.17	-0.049 ± 0.0083
SCZ vs T2D (EUR)	-0.098 ± 0.029	0.009	0.0018	8488.47 ± 483.84	211.65 ± 205.38	1176.33 ± 244.79	-0.24 ± 0.06	-0.076 ± 0.0066

**Fig. 1:**
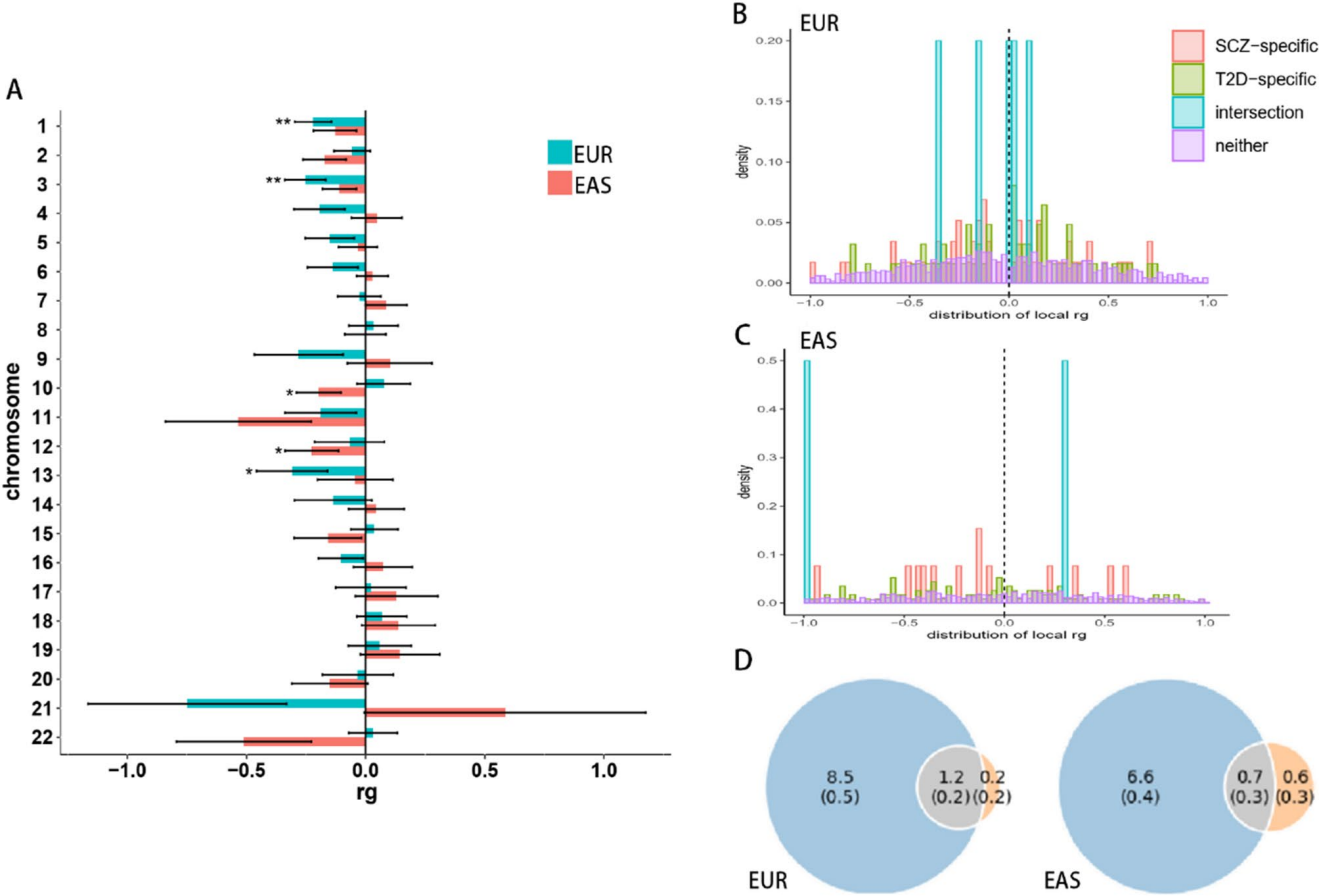
Genetic correlation between SCZ and T2DM. (A) partitioned genetic correlation between SCZ and T2DM in terms of 22 autosomes in both EUR and EAS populations with LDSC method. Mean and standard error bars are shown. (B) and (C) density distribution of local genetic correlation between two disorders identified with HESS method within four different disease-related regions in the EUR and EAS populations. (D) Venn diagrams of unique and common causal DNA loci between SCZ (left blue) and T2DM (right pink) identified with the MiXeR analysis, the estimated quantity of causal variants (in thousands) is upper, its standard error is down

Furthermore, the partitioned genetic correlation analysis results demonstrated that in the EUR samples, chromosomes 1, 3, and 13 had significant correlations (r_g_ = − 0.22, − 0.25, and − 0.31, and *P* = 0.0042, 0.0033, and 0.039, respectively), and in the EAS samples, chromosomes 10 and 2 had significant correlations (r_g_ =- 0.20 and − 0.23, and *P* = 0.035 and 0.044, respectively, **Table S1** and Fig. 1 A). Nevertheless, only chromosomes 1 and 3 in the EUR samples remained Bonferroni significant (*P* < 0.05/2 = 0.025).

The results of the local genetic correlation analysis with HESS showed that in the EUR samples, 157 loci had a correlation with a *P* value less than 0.05; and in the EAS samples there were 23 loci (**Table S2**). Nevertheless, only chr18:51554175–55,213,838 (*P* = 2.03 × 10^− 6^) and chr6:63552888–65,765,742 (*P* = 4.36 × 10^− 6^) in the EUR samples remained Bonferroni significant [P < 0.05/(2 × 1702) = 1.47 × 10^− 5^]. Furthermore, the number of loci containing the GWAS significant SNPs that were specific to SCZ, specific to T2DM, related to both diseases and related to neither were 69, 62, 5 and 1566, respectively, in the EUR samples, and 14, 150, 2 and 1277, respectively, in the EAS samples. Additionally, the genetic correlations of both the SCZ- and T2DM-specific loci largely had negative values, which supported the genome-wide results from the LDSC analysis (Fig. 1B and **C**). SCZ- or T2DM-specific loci, rather than common loci were more likely to have a negative maximum genetic correlation in the EUR samples than those in the EAS samples.

The polygenic overlapping analysis results also supported the negative correlation of SCZ and T2DM effect sizes within the shared causal variants, with ρ = − 0.24 ± 0.057 and − 0.28 ± 0.17, and r_g_ = − 0.049 ± 0.0083 and − 0.076 ± 0.0066 for the EAS and EUR populations, respectively (**Table **1; Fig. 1D). Furthermore, SCZ and T2DM had a low polygenic overlap, sharing only approximately 0.7 K of the 7.9 K causal variants (8.9%) and 1.2 K of the 9.9 K causal variants (12.1%) for the EAS and EUR populations, respectively. However, common causal variants accounted for 85.7% of the T2DM causal variants in EUR populations.

**Fig. 2:**
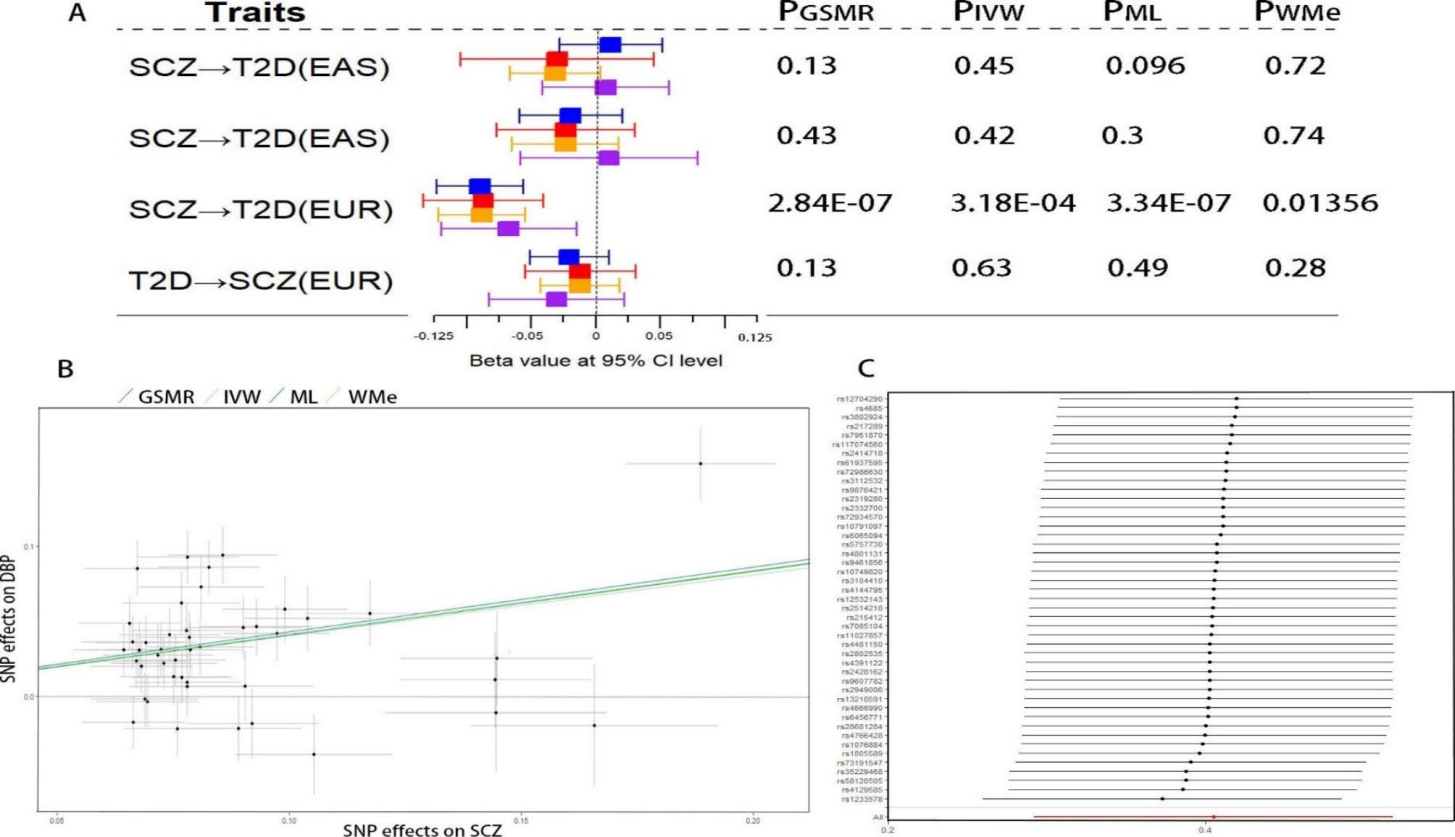
Mendelian Randomization (MR) analyses between SCZ and T2DM/T2DM-related trait of diastolic blood pressure. (A) The dual directional causal effect between SCZ and T2DM in different population with four methods, i.e. GSMR in blue, IVW in red, ML in yellow, WMe in purple. (B) Relationship of 45 instrumental variants’ effects on SCZ and those on DBP (diastolic blood pressure), each dot represents an instrumental variant, its 95% CIs for the estimated effect on SCZ and DBP denote horizontal and vertical lines, respectively, and the lines represent the causal effects of SCZ on DBP in the EUR population with four methods. (C) Sensitive analysis of each instrumental variant’s influence on the estimated causal effects of SCZ on DBP using the IVW method

*Mendelian randomization analysis*The results of the MR analyses based on the four methods (GSMR, IVW, ML, and WMe) indicated that SCZ may have a genetically negative causal effect on T2DM in the EUR samples with 66 instrumental variants (P = 2.84 × 10^− 7^, 3.18 × 10^− 4^, 3.34 × 10^− 7^ and 0.014 for GSMR, IVW, ML and WMe, respectively, Fig. 2 A and **Table S3**). However, the Bonferroni-corrected P value from the WMe method was 0.056. In these analyses, the Mendelian randomization-Egger (MR-Egger) and Mendelian Randomization Pleiotropy RESidual Sum and Outlier (MR-PRESSO) tests did not support the existence of pleiotropic effects biasing the estimates of the causal effects of SCZ on T2DM in the EUR samples (MR Egger intercept, − 0.01; *P* = 0. 23; *P* value for the outlier test∈[0.23,1]).

### Genome-wide cross-trait/ethnic meta-analysis

**Fig. 3:**
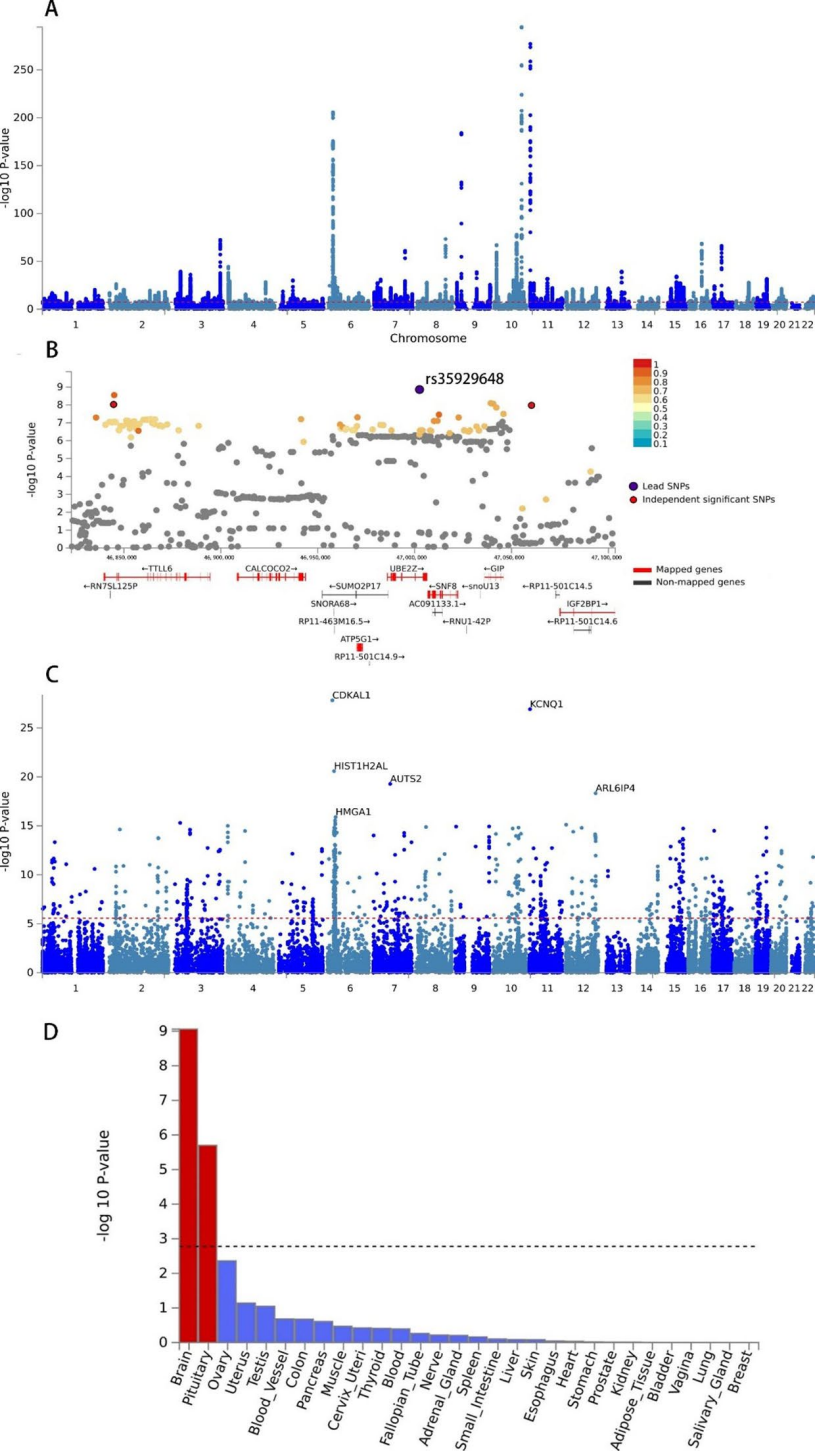
Genome-wide cross-trait/ethnic meta-analysis results of SCZ and T2DM through using CPASSOC methods. A. A representative Manhattan plot of meta-analysis result of combined EAS and EUR population. The x-axis is the chromosomal position of SNPs and the y-axis is the significance of the SNPs (-log10P). B: One representative genomic risk locus containing a novel top independent cross-trait significant SNP (indSigSNPs), i.e. rs35929648. IndSigSNPs are SNPs independent of each other (r2 < 0.6) with P ≤ 5.0 × 10 − 8, top IndSigSNPs are IndSigSNPs with the minimum P value within a 250 kb window. Genomic risk loci were identified by merging IndSigSNPs if they were closer than 500 kb apart. In this genomic risk loci, there are three independent significant SNPs. Each SNP is color-coded based on the highest r2 to one of the independent significant SNPs (indSigSNPs) if that is greater or equal to the r2 threshold of 0.6. Other SNPs (below the r2 of 0.6) are colored in grey. Gene property analysis results. C. Positional genes mapping results with the top 6 genes labelled. D. Tissue specificity of indsigSNPs in GTEx V8 30 tissue types using the MAGMA tool

A total of 24, 627 genome-wide significant SNPs were found with the CPASSOC method, which were located on almost all the autosomes (Fig. 3 A). Furthermore, 1, 313 SNPs were identified that had not been reported as significant variants in either of the previous SCZ or T2DM GWASs (**Figure S2**). Among these 1, 313 SNPs, 25 SNPs were independent variants responsible for the comorbidity of SCZ and T2DM (**Table S4**, Fig. 3B).

### Positional gene mapping

Based on the results from the genome-wide cross-trait/ethnic meta-analysis with the CPASSOC method, a total of 1, 033 common protein-coding genes were mapped. Among them, the top 6 genes were *CDKAL1* (chr6:20,534,688 − 21,232,635, based on GRCh37/hg19), *KCNQ1* (chr11:2,466,238-2,870,340), *HIST1H2AL* (chr6:27,833,095 − 27,833,576), *AUTS2* (chr7:69,063,461 − 70,258,492), *ARL6IP4* (chr12:123,464,780 − 123,467,456) and *HMGA1* (chr6:34,204,650 − 34,214,008, Fig. 3 C). Furthermore, the MAGMA gene property analysis results for tissue specificity demonstrated that the cross-strait associated genes were significantly correlated with the brain and the pituitary gland (*P* = 1.1 × 10^− 9^ and 1.9 × 10^− 6^, respectively, Fig. 3D).

### Cell type-specific analysis

To explore the effective cell types in SCZ and T2DM, the scRNA-Seq dataset was used, including a total of 68, 579 cells and 32, 738 annotated genes. Under the regression model, CD19 + B cells were identified to be potentially trans-ethnic effective cells for SCZ and T2DM **(Figure S3)**.

### Transcriptome-wide cross-trait/ethnicity meta-analysis

With the TWAS data obtained from the FUSION analysis, the ASSET results suggested that 5, 417 genes had combined beta and *P* values (**Table S5**). Among these genes, 61 genes exceeded the Bonferroni significance threshold of *P* < 0.05/5417 = 9.23 × 10^− 6^. The genes *TCF19* and *SNX11* and the lncRNA *NFE2L1-DT* had significantly positive effects on either SCZ, T2D, or both in the EUR or EAS samples, while the lncRNA *RP5_890E165* had significantly negative effects. Among these 61 genes, 33 genes had positive beta values, of which 24 genes (72.7%) were protein-coding ones; and 28 had negative beta values, of which 14 (50%) encoded proteins.

### IPA analysis

**Fig. 4:**
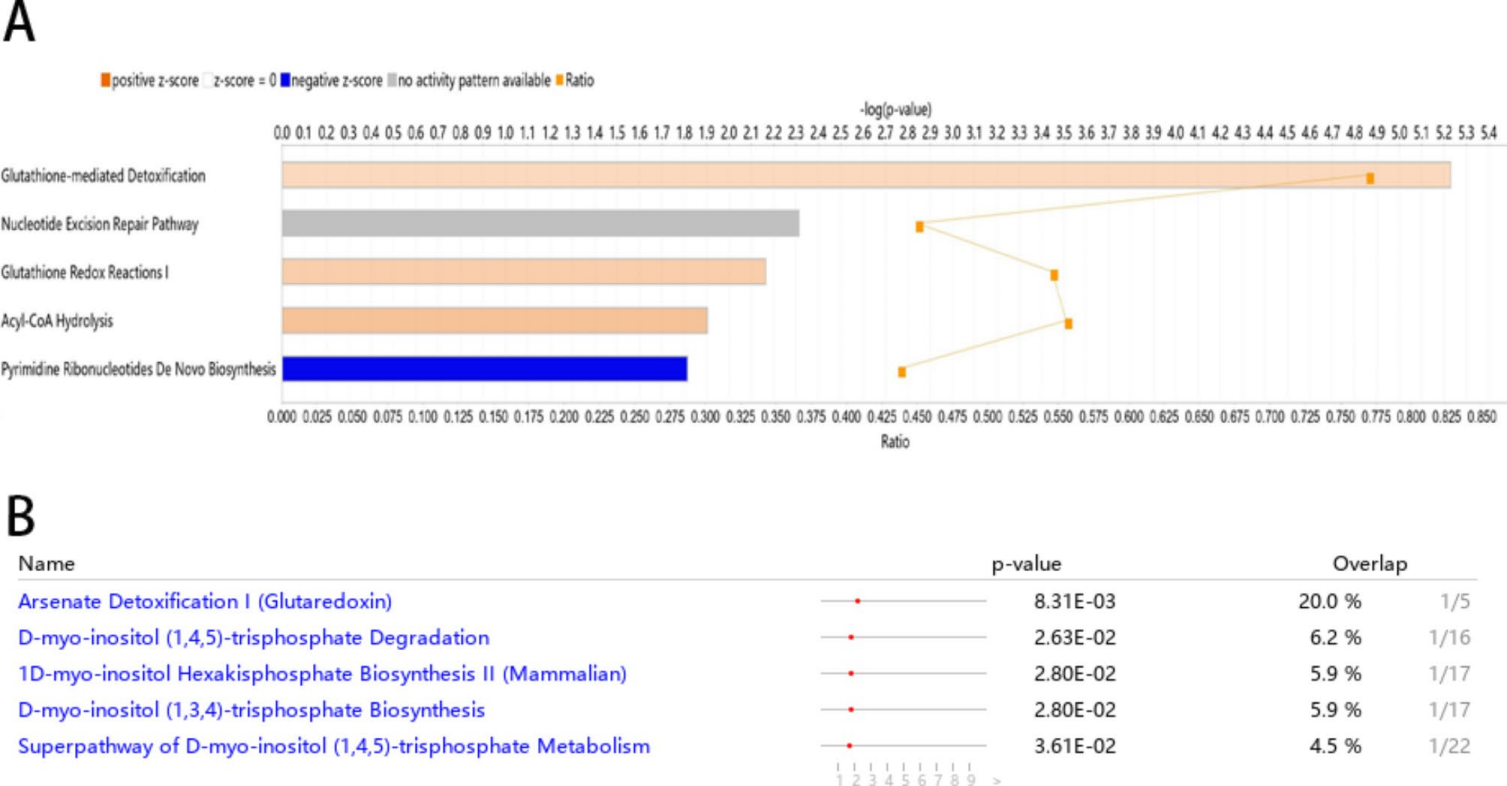
IPA analysis results. (A) with the Beta values of whole mapped genes as input; (B) with the P values of the genes with P < 9.33E-6 (0.05/5359) as input

Among the above 5, 417 genes, 5, 359 genes were mapped to known gene symbols. Using beta values as the input, the whole mapped genes were significantly enriched in the following top 5 canonical pathways: the up-regulated glutathione-mediated detoxification pathway (*P* = 5.89 × 10^− 6^), nucleotide excision repair pathway (*P* = 4.85 × 10^− 3^), glutathione redox reactions I (*P* = 6.84 × 10^− 3^), acyl-CoA hydrolysis (*P* = 1.25 × 10^− 2^) and the down-regulated pyrimidine ribonucleotides de novo biosynthesis pathway (*P = 1.5*3 × 10^− 2^, Fig. 4 A). Using *P* values as the inputs, the genes with *P* values less than the threshold of 9.33 × 10^− 6^ (0.05/5359) were significantly enriched in the following top 5 canonical pathways: arsenate detoxification I (glutaredoxin, *P* = 8.31 × 10^− 3^) and four other pathways associated with D-myo-inositol (1,4,5)-triphosphate metabolism (Fig. 4B).

## Discussion

Here, we employed a series of genomic analysis approaches, which were complementary and leveraged GWAS summary data, to explore the genetic basis for the comorbidity of SCZ and T2DM. Moreover, large-scale trans-ethnic data were used to minimize the effects of potential confounding factors and some Eurocentric bias and to enhance the power of the analysis with regard to identifying trait associations. Finally, additional evidence was reported for the genetic correlation between SCZ and T2DM, and the pituitary gland was identified as a common effective organ for these two diseases. These results suggest that the comorbidity between SCZ and T2DM could be partially attributed to shared effective genes in particular organs rather than to genes in the whole body or to environmental factors alone.

Based on the heritability distributed over many variants with small effects and on the analysis using genome-wide variants instead of variants significantly associated with a disorder [30], we found that SCZ had a significantly negative genetic correlation with T2DM in both the EUR and the EAS populations. Although the negative correlation remained significant only in the EUR samples after Bonferroni correction, there was no significant difference in the genetic correlation between the two populations. Similar results were obtained from the partitioned LDSC analysis of each autosome and the HESS analysis of different loci in each autosome. Next, based on the variants significantly associated with a disorder, which represent a substantial fraction of heritability in many diseases [31], the MiXeR analysis results also supported the negative genetic correlation between the two diseases, and the MR analysis results suggested that in the EUR population, a genetic predisposition to SCZ tended to prevent T2DM. The effective gene analysis results suggested that more non-protein-coding genes, such as lncRNAs, were likely to take part in such prevention in the pituitary gland.

The reported Bonferroni-significant negative whole-genome correlation between SCZ and T2DM in Europeans was not observed in previous studies, in which nonsignificant negative whole-genome correlation was found with EUR sample sizes close to 140,000 [7, 8]. However, a forthcoming article also found a significant negative whole-genome correlation on about 7 times more EUR subjects than those in previous studies using the same method of LDSC [9]. This inconsistence may be because more recent GWAS data are used and a larger sample size may enhance the power of the correlation analysis. Furthermore, at the autosomes and loci level, we found multiple regions of positive and negative correlation between T2D and SCZ, a large sample size in the LDSC analysis may eliminate the biased estimates to null and provide more reliable overall analysis results[32].

The reported genetic predisposition to SCZ with the tendency to prevent T2DM is incompatible with the epidemiological data indicating an elevated risk of T2DM in SCZ[3, 4]. SCZ patients may have some lifestyle factors that predispose them to T2DM, such as an unusual diet and easy access to antipsychotics, which may outweigh the presence of the moderate genetic preventive factors and initiate T2DM by modulating non-protein-coding genes. It has been speculated that SCZ risk alleles should be naturally selected for elimination as patients with SCZ have reduced fertility and increased mortality. Conversely, the MiXeR analysis results demonstrated that the SCZ causal variants were of a larger number than those of T2DM and that the majority of T2DM causal variants overlapped with those for SCZ. Interestingly, in the EUR samples, single disorder-specific genomic regions rather than common regions were found to be more likely to cause the maximum negative genetic correlation than was the case in the EAS samples. According to the morphological evolution standpoint that negative genetic correlations between traits may result in a substantial change in morphology [33], there are microevolutionary responses to the negative genetic correlation between SCZ and T2DM. These suggest that the impairment of molecular modulation in certain organs rather than in the whole body is responsible for the comorbidity of SCZ and T2DM.

Through genome-wide cross-trait/ethnic meta-analysis and positional gene mapping, the top six genes of CDKAL1, KCNQ1, HIST1H2AL, AUTS2, ARL6IP4 and HMGA1 are mainly located on chromosomes 6, 7, 11 and 12, while the top SNPs in chromosomes 9 and 10 are located on the non-protein-coding region. The top one gene CDKAL1 encoding a member of the methylthiotransferase family, a subfamily of the radical S-adenosylmethionine (SAM) superfamily, has been reported be involved in the susceptibility to T2DM in Europeans and Japanese [34, 35] and to bipolar disorder in Europeans[36]. Recent research demonstrates that Cdkal1 is necessary for normal mitochondrial morphology by regulating mitochondrial activity. HIST1H2AL located in a histone gene cluster region on chromosome 6p, encodes one of the core histone proteins, Histone H2A type. Histone variants involved in nucleosome composition and histone modification are important for neurodevelopment and are related to the susceptibility of psychiatric disorders[37, 38]. HMGA1 encoding a nonhistone architectural transcription factor is involved in fundamental cellular processes by regulating chromatin structure and multiple gene expression including the insulin receptor (INSR) and Forkhead box protein O1 (FoxO1)[39]. INSR is a master regulatory factor for insulin action and glucose homeostasis, and FoxO1 is a critical regulatory factor for gluconeogenesis and glycogenolysis[40]. AUTS2, i.e. autism-susceptibility-gene-2, encodes an activator of transcription and regulates neurodevelopment. AUTS2 variants can cause a neurodevelopmental and somatic malformation with diverse phenotypes[41]. KCNQ1 located on chromosome 11q, encodes a member of voltage-gated potassium channel subfamily that can affect cardiac and neuronal action potentials. KCNQ1 has been reported to be associated with SCZ and T2D[42, 43]. ARL6IP4 encodes ADP ribosylation factor like GTPase 6 (ARL6) interacting protein 4 is predicted to be involved in RNA splicing and mRNA processing and has been reported to be associated with SCZ[44]. Although the roles of ARL6IP4 is unknown, ARL6 is known to regulate intracellular protein traffic[45].

Through genome-wide cross-trait/ethnic meta-analysis and integration analysis of tissue-specific gene expression profiles, the pituitary gland was identified as the effective organ for both SCZ and T2DM. Furthermore, the molecular metabolism pathways related to glutathione mediated arsenate detoxification and D-myo-inositol-trisphosphate metabolism were identified as potential basic molecular modulation mechanisms. The pituitary gland is a highly plastic system that can integrate the information from both external and internal environments and maintain homeostasis by the rhythmic secretion of key hormones, such as adrenocorticotrophic hormone and growth hormone, in pulse manner in all vertebrates[46]. The pituitary is called the master gland since it controls the function of most other endocrine glands, such as the thyroid and adrenal cortex [47]. In humans, the pituitary can be divided into three anatomically and developmentally distinct constituent parts, i.e. the neurohypophysis (posterior lobe), the adenohypophysis (anterior lobe) and the intermediate lobe[48]. And pituitary cells have been found to organize in tightly wired networks in both homo and heterotypic manners and communicate with each other[48]. Thus, the pituitary gland can quickly integrate the hypothalamic and systemic stimuli and optimize its function. It is a central part of the hypothalamic-pituitary gland-adrenal (HPA) axis, which is an important neuroendocrine system with a fundamental role in physiological adaptive responses to stressors [49]. Cumulative psychiatric stress may induce allostatic load, and exert additional systemic and detrimental effects on neuroendocrine dysfunction, ultimately leading to the onset of T2DM.

Furthermore, it has been reported that chronic inorganic arsenic exposure can lead to neurobehavioral alterations and T2DM [50, 51]. Although our previous study did not find a significant association of serum arsenic concentration with the risk of SCZ [1], we found that the *GSTM1* (glutathione S-transferase Mu-1 (GSTM1) gene) null genotype had a risk ratio of 1.14 for SCZ [52]; *GSTM1* is involved in arsenic metabolism and detoxification in humans [53]. D-myo-inositol-trisphosphate is a second messenger and mobilizes calcium (Ca2+). SCZ involves an abnormality in second messenger precursor availability [54] and thus is characterized by reduced D-myo-inositol-trisphosphate levels. However, inositol supplementation is an effective and safe strategy for treating T2DM [55].

CD19, i.e. Cluster of Differentiation 19, is expressed in all B lineage cells in humans and is a B-Lymphocyte surface biomarker[56]. CD19 + B cells are so important that they can mediate immune response and regulation by the activation of T cells, the release of antibodies and the secretion of cytokines[57]. Through producing antigen-specific antibody, CD19 + B cells can build the first line of defense against exogenous antigens and further facilitate phagocytosis for destruction and antigen-presentation. During the process, polyclonal B cells may be produced through a mechanism called bystander activation, which can enhance the excessive inflammatory response and self-destruct normal cells[58]. B-cells have been suggested to be potentially therapeutic targets for SCZ and T2D[57, 59]. However, no evidence is found for altered numbers of the CD19 + B cells in blood of patients with SCZ except the B-cell related cytokines and certain autoantibodies[59]. And compared to obese subjects with non-T2D, obese subjects with T2D have been found to have several B cell defects in blood, including lower IL-10 production and ineffective antibody response to new antigens, but present much higher levels of polyclonal activation and antibody secretion[60]. However, it is unclear if these findings result from T2D development or contribute to T2D pathogenesis in obesity. Although our study has identified CD19 + B cells as potentially trans-ethnic effective cells for SCZ and T2DM, a detailed mechanistic framework requires more actual laboratory work for explanation.

The main limitations of this study as follows: First, the lack of actual laboratory work validated the findings. Nevertheless, the current work strived to obtain the validated findings using a set of complementary methods to perform large-scale analyses at different levels. Second, the stringent Bonferroni correction led to some findings not reaching the threshold of significance. However, this is an effective method of controlling the risk of a type I statistical error. Third, the lack of individual data made it impossible to stratify the analysis by the severity of SCZ. Forth, only SNPs with MAF > 5% were included in these genetic analyses. These are common limitations to approaches based on summary GWAS data. Finally, no T2DM- or SCZ-related traits were analyzed here to provide more evidence. Our future research will investigate the corresponding traits.

The current study identified the pituitary gland as a common effective organ for both T2DM and SCZ, despite T2DM showing a negative genetic correlation with SCZ. Further research may consider T2DM-related glycaemic/lipid/blood pressure traits, including two-hour glucose, fasting glucose, fasting insulin, proinsulin, glycated hemoglobin A1c (HBA1c), low-density lipoprotein cholesterol (LDL), high-density lipoprotein cholesterol (HDL), total cholesterol, triglycerides, systolic blood pressure, diastolic blood pressure, and pulse pressure, and may also consider other mental disorders, such as depression. Future spatial transcriptomics studies using fresh samples may help to verify our findings and to provide new insights into the comorbidity of T2DM and SCZ.

## Conclusion

In summary, a negative genetic correlation exists between SCZ and T2DM at the whole genome, autosome, locus and causal variant levels, which suggests that shared effective genes in a particular organ may contribute to the comorbidity of SCZ and T2DM. The pituitary gland was identified as a common effective organ for T2DM and SCZ, in which more non-protein-encoding effective genes, such as lncRNAs, may be responsible for the identified negative genetic correlations. This highlights the importance of molecular metabolism and neuroendocrine modulation in the pituitary gland, which may be responsible for T2DM in SCZ patients.

## Electronic supplementary material

Below is the link to the electronic supplementary material.

Supplementary Material 1

## Data Availability

The data are available from the corresponding author upon reasonable request.

## References

[1] Cai L, Chen T, Yang J, Zhou K, Yan X, Chen W, Sun L, Li L, Qin S, Wang P (2015). Serum trace element differences between Schizophrenia patients and controls in the Han Chinese population. Sci Rep.

[2] Meigs JB (2019). The Genetic Epidemiology of Type 2 Diabetes: Opportunities for Health Translation. Curr Diab Rep.

[3] Holt RI, Mitchell AJ (2015). Diabetes mellitus and severe mental illness: mechanisms and clinical implications. Nat Rev Endocrinol.

[4] Mizuki Y, Sakamoto S, Okahisa Y, Yada Y, Hashimoto N, Takaki M, Yamada N (2021). Mechanisms Underlying the Comorbidity of Schizophrenia and Type 2 Diabetes Mellitus. Int J Neuropsychopharmacol.

[5] Pillinger T, Beck K, Gobjila C, Donocik JG, Jauhar S, Howes OD (2017). Impaired Glucose Homeostasis in First-Episode Schizophrenia: A Systematic Review and Meta-analysis. JAMA Psychiatry.

[6] Rajkumar AP, Horsdal HT, Wimberley T, Cohen D, Mors O, Borglum AD, Gasse C (2017). Endogenous and Antipsychotic-Related Risks for Diabetes Mellitus in Young People With Schizophrenia: A Danish Population-Based Cohort Study. Am J Psychiatry.

[7] Bulik-Sullivan B, Finucane HK, Anttila V, Gusev A, Day FR, Loh PR, ReproGen C, Psychiatric Genomics C, Control C, Duncan L, Genetic Consortium for Anorexia Nervosa of the Wellcome Trust Case (2015). An atlas of genetic correlations across human diseases and traits. Nat Genet.

[8] Hackinger S, Prins B, Mamakou V, Zengini E, Marouli E, Brcic L, Serafetinidis I, Lamnissou K, Kontaxakis V, Dedoussis G (2018). Evidence for genetic contribution to the increased risk of type 2 diabetes in schizophrenia. Transl Psychiatry.

[9] Perry BI, Bowker N, Burgess S, Wareham NJ, Upthegrove R, Jones PB, Langenberg C, Khandaker GM (2022). Evidence for Shared Genetic Aetiology Between Schizophrenia, Cardiometabolic, and Inflammation-Related Traits: Genetic Correlation and Colocalization Analyses. Schizophr Bull Open.

[10] Lam M, Chen CY, Li Z, Martin AR, Bryois J, Ma X, Gaspar H, Ikeda M, Benyamin B, Brown BC (2019). Comparative genetic architectures of schizophrenia in East Asian and European populations. Nat Genet.

[11] Schizophrenia Working Group of the Psychiatric Genomics (2014). Biological insights from 108 schizophrenia-associated genetic loci. Nature.

[12] Mahajan A, Taliun D, Thurner M, Robertson NR, Torres JM, Rayner NW, Payne AJ, Steinthorsdottir V, Scott RA, Grarup N (2018). Fine-mapping type 2 diabetes loci to single-variant resolution using high-density imputation and islet-specific epigenome maps. Nat Genet.

[13] Spracklen CN, Horikoshi M, Kim YJ, Lin K, Bragg F, Moon S, Suzuki K, Tam CHT, Tabara Y, Kwak SH (2020). Identification of type 2 diabetes loci in 433,540 East Asian individuals. Nature.

[14] Genomes Project C, Auton A, Brooks LD, Durbin RM, Garrison EP, Kang HM, Korbel JO, Marchini JL, McCarthy S, McVean GA, Abecasis GR (2015). A global reference for human genetic variation. Nature.

[15] Shi H, Kichaev G, Pasaniuc B (2016). Contrasting the Genetic Architecture of 30 Complex Traits from Summary Association Data. Am J Hum Genet.

[16] Berisa T, Pickrell JK (2016). Approximately independent linkage disequilibrium blocks in human populations. Bioinformatics.

[17] Frei O, Holland D, Smeland OB, Shadrin AA, Fan CC, Maeland S, O’Connell KS, Wang Y, Djurovic S, Thompson WK (2019). Bivariate causal mixture model quantifies polygenic overlap between complex traits beyond genetic correlation. Nat Commun.

[18] Yang J, Lee SH, Goddard ME, Visscher PM (2011). GCTA: a tool for genome-wide complex trait analysis. Am J Hum Genet.

[19] Smith GD, Ebrahim S (2003). ’Mendelian randomization’: can genetic epidemiology contribute to understanding environmental determinants of disease?. Int J Epidemiol.

[20] Verbanck M, Chen CY, Neale B, Do R (2018). Detection of widespread horizontal pleiotropy in causal relationships inferred from Mendelian randomization between complex traits and diseases. Nat Genet.

[21] Zhu X, Feng T, Tayo BO, Liang J, Young JH, Franceschini N, Smith JA, Yanek LR, Sun YV, Edwards TL (2015). Meta-analysis of correlated traits via summary statistics from GWASs with an application in hypertension. Am J Hum Genet.

[22] de Leeuw CA, Mooij JM, Heskes T, Posthuma D (2015). MAGMA: generalized gene-set analysis of GWAS data. PLoS Comput Biol.

[23] Watanabe K, Taskesen E, van Bochoven A, Posthuma D (2017). Functional mapping and annotation of genetic associations with FUMA. Nat Commun.

[24] Zheng GX, Terry JM, Belgrader P, Ryvkin P, Bent ZW, Wilson R, Ziraldo SB, Wheeler TD, McDermott GP, Zhu J (2017). Massively parallel digital transcriptional profiling of single cells. Nat Commun.

[25] Gusev A, Ko A, Shi H, Bhatia G, Chung W, Penninx BW, Jansen R, de Geus EJ, Boomsma DI, Wright FA (2016). Integrative approaches for large-scale transcriptome-wide association studies. Nat Genet.

[26] Bhattacharjee S, Rajaraman P, Jacobs KB, Wheeler WA, Melin BS, Hartge P, GliomaScan C, Yeager M, Chung CC, Chanock SJ, Chatterjee N (2012). A subset-based approach improves power and interpretation for the combined analysis of genetic association studies of heterogeneous traits. Am J Hum Genet.

[27] Cai L, Huang T, Su J, Zhang X, Chen W, Zhang F, He L, Chou KC (2018). Implications of Newly Identified Brain eQTL Genes and Their Interactors in Schizophrenia. Mol Ther Nucleic Acids.

[28] Bulik-Sullivan BK, Loh PR, Finucane HK, Ripke S, Yang J, Genomics C, Patterson N, Daly MJ, Price AL, Neale BM, Schizophrenia Working Group of the Psychiatric (2015). LD Score regression distinguishes confounding from polygenicity in genome-wide association studies. Nat Genet.

[29] Yang Y, Musco H, Simpson-Yap S, Zhu Z, Wang Y, Lin X, Zhang J, Taylor B, Gratten J, Zhou Y (2021). Investigating the shared genetic architecture between multiple sclerosis and inflammatory bowel diseases. Nat Commun.

[30] Visscher PM, Brown MA, McCarthy MI, Yang J (2012). Five years of GWAS discovery. Am J Hum Genet.

[31] Voight BF, Peloso GM, Orho-Melander M, Frikke-Schmidt R, Barbalic M, Jensen MK, Hindy G, Holm H, Ding EL, Johnson T (2012). Plasma HDL cholesterol and risk of myocardial infarction: a mendelian randomisation study. Lancet.

[32] Shi H, Mancuso N, Spendlove S, Pasaniuc B (2017). Local Genetic Correlation Gives Insights into the Shared Genetic Architecture of Complex Traits. Am J Hum Genet.

[33] Norry FM, Vilardi JC, Hasson E (2000). Negative genetic correlation between traits of the Drosophila head, and interspecific divergence in head shape. Heredity (Edinb).

[34] Steinthorsdottir V, Thorleifsson G, Reynisdottir I, Benediktsson R, Jonsdottir T, Walters GB, Styrkarsdottir U, Gretarsdottir S, Emilsson V, Ghosh S (2007). A variant in CDKAL1 influences insulin response and risk of type 2 diabetes. Nat Genet.

[35] Omori S, Tanaka Y, Takahashi A, Hirose H, Kashiwagi A, Kaku K, Kawamori R, Nakamura Y, Maeda S (2008). Association of CDKAL1, IGF2BP2, CDKN2A/B, HHEX, SLC30A8, and KCNJ11 with susceptibility to type 2 diabetes in a Japanese population. Diabetes.

[36] Nurnberger JI, Koller DL, Jung J, Edenberg HJ, Foroud T, Guella I, Vawter MP, Kelsoe JR (2014). Psychiatric Genomics Consortium Bipolar G: Identification of pathways for bipolar disorder: a meta-analysis. JAMA Psychiatry.

[37] Shi J, Levinson DF, Duan J, Sanders AR, Zheng Y, Pe’er I, Dudbridge F, Holmans PA, Whittemore AS, Mowry BJ (2009). Common variants on chromosome 6p22.1 are associated with schizophrenia. Nature.

[38] Yamagata H, Uchida S, Matsuo K, Harada K, Kobayashi A, Nakashima M, Nakano M, Otsuki K, Abe-Higuchi N, Higuchi F (2017). Identification of commonly altered genes between in major depressive disorder and a mouse model of depression. Sci Rep.

[39] Chiefari E, Foti DP, Sgarra R, Pegoraro S, Arcidiacono B, Brunetti FS, Greco M, Manfioletti G, Brunetti A (2018). Transcriptional Regulation of Glucose Metabolism: The Emerging Role of the HMGA1 Chromatin Factor. Front Endocrinol (Lausanne).

[40] Foti D, Chiefari E, Fedele M, Iuliano R, Brunetti L, Paonessa F, Manfioletti G, Barbetti F, Brunetti A, Croce CM (2005). Lack of the architectural factor HMGA1 causes insulin resistance and diabetes in humans and mice. Nat Med.

[41] Gao Z, Lee P, Stafford JM, von Schimmelmann M, Schaefer A, Reinberg D (2014). An AUTS2-Polycomb complex activates gene expression in the CNS. Nature.

[42] Al-Shammari MS, Al-Ali R, Al-Balawi N, Al-Enazi MS, Al-Muraikhi AA, Busaleh FN, Al-Sahwan AS, Al-Elq A, Al-Nafaie AN, Borgio JF (2017). Type 2 diabetes associated variants of KCNQ1 strongly confer the risk of cardiovascular disease among the Saudi Arabian population. Genet Mol Biol.

[43] Bruce HA, Kochunov P, Paciga SA, Hyde CL, Chen X, Xie Z, Zhang B, Xi HS, O’Donnell P, Whelan C (2017). Potassium channel gene associations with joint processing speed and white matter impairments in schizophrenia. Genes Brain Behav.

[44] Pouget JG, Genomics C, Han B, Wu Y, Mignot E, Ollila HM, Barker J, Spain S, Dand N, Trembath R, Schizophrenia Working Group of the Psychiatric (2019). Cross-disorder analysis of schizophrenia and 19 immune-mediated diseases identifies shared genetic risk. Hum Mol Genet.

[45] Jin H, White SR, Shida T, Schulz S, Aguiar M, Gygi SP, Bazan JF, Nachury MV (2010). The conserved Bardet-Biedl syndrome proteins assemble a coat that traffics membrane proteins to cilia. Cell.

[46] Le Tissier P, Campos P, Lafont C, Romano N, Hodson DJ, Mollard P (2017). An updated view of hypothalamic-vascular-pituitary unit function and plasticity. Nat Rev Endocrinol.

[47] Bhattacharya S, Kalra S, Dutta D, Khandelwal D, Singla R (2020). The Interplay Between Pituitary Health and Diabetes Mellitus - The Need for ‘Hypophyseo-Vigilance’. Eur Endocrinol.

[48] Santiago-Andres Y, Golan M, Fiordelisio T (2020). Functional Pituitary Networks in Vertebrates. Front Endocrinol (Lausanne).

[49] Nicolaides NC, Kyratzi E, Lamprokostopoulou A, Chrousos GP, Charmandari E (2015). Stress, the stress system and the role of glucocorticoids. Neuroimmunomodulation.

[50] Vahidnia A, van der Voet GB, de Wolff FA (2007). Arsenic neurotoxicity–a review. Hum Exp Toxicol.

[51] Islam R, Khan I, Hassan SN, McEvoy M, D’Este C, Attia J, Peel R, Sultana M, Akter S, Milton AH (2012). Association between type 2 diabetes and chronic arsenic exposure in drinking water: a cross sectional study in Bangladesh. Environ Health.

[52] Cai L, Cai MH, Wang MY, Xu YF, Chen WZ, Qin SY, Wan CL, He L (2015). Meta-Analysis-Based Preliminary Exploration of the Connection between ATDILI and Schizophrenia by GSTM1/T1 Gene Polymorphisms. PLoS ONE.

[53] Steinmaus C, Moore LE, Shipp M, Kalman D, Rey OA, Biggs ML, Hopenhayn C, Bates MN, Zheng S, Wiencke JK, Smith AH (2007). Genetic polymorphisms in MTHFR 677 and 1298, GSTM1 and T1, and metabolism of arsenic. J Toxicol Environ Health A.

[54] Shimon H, Sobolev Y, Davidson M, Haroutunian V, Belmaker RH, Agam G (1998). Inositol levels are decreased in postmortem brain of schizophrenic patients. Biol Psychiatry.

[55] Pintaudi B, Di Vieste G, Bonomo M (2016). The Effectiveness of Myo-Inositol and D-Chiro Inositol Treatment in Type 2 Diabetes. Int J Endocrinol.

[56] Martin P, Santon A, Bellas C (2004). Neural cell adhesion molecule expression in plasma cells in bone marrow biopsies and aspirates allows discrimination between multiple myeloma, monoclonal gammopathy of uncertain significance and polyclonal plasmacytosis. Histopathology.

[57] DeFuria J, Belkina AC, Jagannathan-Bogdan M, Snyder-Cappione J, Carr JD, Nersesova YR, Markham D, Strissel KJ, Watkins AA, Zhu M (2013). B cells promote inflammation in obesity and type 2 diabetes through regulation of T-cell function and an inflammatory cytokine profile. Proc Natl Acad Sci U S A.

[58] Moir S, Fauci AS (2013). Insights into B cells and HIV-specific B-cell responses in HIV-infected individuals. Immunol Rev.

[59] van Mierlo HC, Broen JCA, Kahn RS, de Witte LD (2019). B-cells and schizophrenia: A promising link or a finding lost in translation?. Brain Behav Immun.

[60] Zhai X, Qian G, Wang Y, Chen X, Lu J, Zhang Y, Huang Q, Wang Q (2016). Elevated B Cell Activation is Associated with Type 2 Diabetes Development in Obese Subjects. Cell Physiol Biochem.

