# Supplementary material for: Evidence that the pituitary gland connects type 2 diabetes mellitus and schizophrenia based on large-scale trans-ethnic genetic analyses
Source: J Transl Med. 2022 Nov 3;20:501. doi: 10.1186/s12967-022-03704-0 (PMC9632150; doi:10.1186/s12967-022-03704-0)
Supplement: Supplementary file 1 — Supplementary Material 1 [file 12967_2022_3704_MOESM1_ESM.docx]

**Supplementary Materials**

**INDEX**

**1 Data and Methods**

1.1 GWAS dataset description and quality control

1.2 LD (linkage disequilibrium) score regression

1.3 Local genetic correlation analysis

1.4 Polygenic overlap analysis

1.5 Mendelian randomization (MR) analysis

1.6 Genome-wide cross-trait/ethnic meta-analysis

1.7 Positional gene mapping within the platform FUMA

1.8 MAGMA gene property analysis for tissue specificity and Cell type-specificity

**2 References**

**3 Figure S1**. Flowchart of statistical analyses performed in the current study. Yellow background denotes main procedure, orange background denotes main dataset

**4 Figure S2**. Venn diagrams of genome-wide significant SNPs identified with both GWCTM methods and those identified in either GWAS of SCZ or T2D

**5 Figure S3.** Cell type specific analysis with MAGMA

**6 Table S1**. Partitioned genetic correlation in term of autosomes estimated through using LDSC method

**7 Table S2.** Local genetic correlation estimated through using HESS method

**8 Table S3**. 66 instrumental variables for the MR analysis of the causal effect of SCZ on T2D in EUR population

**9 Table S4.** Independent significant SNPs identified by the CPASSOC method

**10 Table S5.** Effective genes (P _Bonferroni_ <9.23E-6) identified in pituitary through using the Transcriptome-wide Cross-trait/ethnic meta-analysis

**1 Data and Methods**

**1.1 GWAS dataset description**

**Schizophrenia (SCZ) for East Asian population | Max Lam et al., 2019**

The SCZ_EAS GWAS dataset includes 22 778 cases and 35 362 controls from 20 cohorts (1) as following table:

| No. | Cohorts | Case | Control | Array | Population |
| --- | --- | --- | --- | --- | --- |
| 1 | IMH-1 | 856 | 946 | I_1M | Han Chinese |
| 2 | IMH-2 | 766 | 913 | I_OZH | Han Chinese |
| 3 | HNK-1 | 476 | 2018 | I_610 | Han Chinese |
| 4 | JPN-1 | 547 | 540 | A_SNP5.0 | Japanese |
| 5 | BIX-1 | 1045 | 2272 | A_SNP6.0 | Han Chinese |
| 6 | BIX-2 | 1021 | 1001 | A_SNP6.0 | Han Chinese |
| 7 | BIX-3 | 489 | 679 | A_SNP6.0 | Han Chinese |
| 8 | XJU-1 | 1846 | 947 | I_OZH | Han Chinese |
| 9 | UMC-1 | 2260 | 2241 | I_Psyc | Han Chinese |
| 10 | UWA-1 | 988 | 1001 | I_Psyc | Indonesia |
| 11 | BJM-1 | 1312 | 1987 | I_OZH | Han Chinese |
| 12 | TAI-1 | 1109 | 1109 | I_Psyc | Han Chinese |
| 13 | TAI-2 | 590 | 590 | I_Psyc | Han Chinese |
| 14 | KOR-1 | 687 | 492 | A_KB | Korean |
| 15 | SIX-1 | 192 | 47 | I_Psyc | Han Chinese |
| 16 | BIX-4 | 399 | 478 | I_GSA | Han Chinese |
| 17 | BJM-2 | 746 | 1599 | I_610 | Han Chinese |
| 18 | BJM-3 | 1595 | 1447 | I_660W | Han Chinese |
| 19 | BJM-4 | 710 | 680 | I_OZH | Han Chinese |
| 20 | BIX-5 | 5144 | 14375 | A_SNP6.0, A_CHB1, I_1M | Han Chinese |

Among these samples, TAI-1 and TAI-2 who are diagnosed as SCZ according to DIGS had parent-offspring trios and were processed as case/pseudo-controls. other samples are diagnosed as SCZ according to DSM-IV. Each study undertook quality control procedures to exclude variants with: (1) call rate below 95%; (2) monomorphic ones; (3) missing rate differences greater than 2% between cases and controls; (4) in violation of Hardy-Weinberg equilibrium (P < 10−6 for controls or P < 10−10 for cases); and to exclude subjects with: (1) call rate below 98%; (2) inbred coefficient above 0.2 and below −0.2; (3) mismatch in reported sex and chromosome X computed sex.

**SCZ for White European population |** [**Schizophrenia Working Group of the Psychiatric Genomics Consortium**](https://pubmed.ncbi.nlm.nih.gov/?size=50&term=Schizophrenia+Working+Group+of+the+Psychiatric+Genomics+Consortium%5BCorporate+Author%5D) **2014**

This SCZ_EUR subjects includes 33 640 cases and 43 456 control from 49 studies (2) as following table:

Genotypes from all studies were processed by the PGC using unified quality control procedures followed by imputation of SNPs and insertion-deletions using the 1000 Genomes Project reference panel. The quality was controlled as imputation INFO score ≥ 0.6, MAF ≥ 0.01, and successfully imputed in ≥ 20 samples).

| No. | Studies | Cases | Controls | Array |
| --- | --- | --- | --- | --- |
| 1 | Umeå, Sweden | 341 | 577 | omni |
| 2 | Umeå, Sweden | 193 | 704 | omni |
| 3 | Norway (TOP) | 377 | 403 | A6.0 |
| 4 | Edinburgh, UK | 367 | 284 | A6.0 |
| 5 | Denmark | 876 | 871 | I650 |
| 6 | Seven countries (PEIC, WTCCC2) | 574 | 1812 | I1M |
| 7 | Spain (PEIC, WTCCC2) | 150 | 236 | I1M |
| 8 | New York, US & Israel | 325 | 139 | A6.0 |
| 9 | Ireland | 264 | 839 | A6.0 |
| 10 | Ireland (WTCCC2) | 1291 | 1006 | A6.0 |
| 11 | Germany (GRAS) | 1067 | 1169 | AXI |
| 12 | Estonia (EGCUT) | 234 | 1152 | omni |
| 13 | J&J, Roche cases, EGCUT controls | 347 | 310 | I317 |
| 14 | J&J, Roche cases, EGCUT controls | 636 | 636 | I317 |
| 15 | J&J, Roche cases, EGCUT controls | 256 | 130 | I610 |
| 16 | J&J, Roche cases, EGCUT controls | 1154 | 2310 | I1M |
| 17 | US, Australia (MGS) | 2638 | 2482 | A6.0 |
| 18 | London, UK | 509 | 485 | A6.0 |
| 19 | Sweden (Hubin) | 265 | 319 | omni |
| 20 | Bulgaria | 195 | 608 | A6.0 |
| 21 | Canada (Toronto) -US(Lilly)-US (MIGen) | 526 | 1644 | A6.0 |
| 22 | Israel | 894 | 1594 | I1M |
| 23 | Six countries, WTCCC controls | 157 | 245 | I550 |
| 24 | New York, US | 190 | 190 | A500 |
| 25 | Australia | 456 | 287 | I650 |
| 26 | Cardiff, UK | 396 | 284 | A500 |
| 27 | UK (CLOZUK) | 3426 | 4085 | I1M |
| 28 | UK (CLOZUK) | 2105 | 1975 | omni |
| 29 | Netherlands | 700 | 607 | I550 |
| 30 | Finland | 186 | 929 | I317 |
| 31 | Finnish | 360 | 1082 | I550 |
| 32 | Portugal | 346 | 215 | A6.0 |
| 33 | Boston, US (CIDAR) | 67 | 65 | omni |
| 34 | Pfizer | 662 | 1172 | I550 |
| 35 | Bonn/Mannheim, Germany | 1773 | 2161 | ILMN* |
| 36 | Munich, Germany | 421 | 312 | I317 |
| 37 | Aberdeen, UK | 719 | 697 | A6.0 |
| 38 | US (CATIE) | 397 | 203 | A500 |
| 39 | Sweden (sw1) | 215 | 210 | A5.0 |
| 40 | Sweden (sw234) | 1980 | 2274 | A6.0 |
| 41 | Sweden (sw5) | 1764 | 2581 | omni |
| 42 | Sweden (sw6) | 975 | 1145 | omni |
| 43 | Cardiff, UK (CogUK) | 530 | 678 | omni |
| 44 | NIMH CBDB | 133 | 269 | O25 |
| 45 | NIMH CBDB | 497 | 389 | I550 |
| 46 | Denmark | 471 | 456 | I650 |
| 47 | Bulgaria | 649 | 649 | A6.0 |
| 48 | Six countries | 516 | 516 | I650 |
| 49 | Bulgaria | 70 | 70 | omni |

**Type Ⅱ diabetes (T2D) for East Asian population | Spracklen et al. 2020**

This T2D_EAS dataset totally contains 77,418 cases and 356,122 controls from three biobanks (CKB, KBA and BBJ) and 20 GWAS that have participated in the Asian Genetic Epidemiology Network (AGEN), a consortium of genetic epidemiology studies of T2D and related traits conducted in individuals of East Asian ancestry, and the Diabetes Meta-analysis of Trans-ethnic Association Studies (DIAMANTE), a consortium examining the genetic contribution to T2D across diverse ancestry populations (3). Diagnosis of diabetes was based on fasting plasma glucose, glycosylated hemoglobin (HbA1c), current taking of antidiabetic medication, and/or history of diabetes treatment. Given T2D case definitions across cohorts differ, cases of type 1 diabetes (T1D) and maturity onset diabetes of the young (MODY) may possibly be included. Samples are genotyped on either commercially available or customized Affymetrix or Illumina genome-wide genotyping arrays. Array quality control criteria implemented within each study, including variant call rate and Hardy-Weinberg equilibrium. Each study adopted a uniform protocol for pre-imputation quality checks and excluded variants with: i) mismatched chromosomal positions or alleles not present in the reference panel; ii) ambiguous alleles (AT/CG) with minor allele frequency (MAF) >40% in the reference panel; or iii) absolute allele frequency differences >20% compared to East Asian-specific allele frequencies. The genotype scaffold for each study was then imputed to the 1000G Phase 1 or 3 reference panel using minimac3 or IMPUTEv2.

**T2D for White European population | Mahajan et al. 2018**

This "DIAMANTE (European) T2D GWAS" dataset contains 74,124 T2D cases and 824,006 controls from 32 cohorts (4) as following table:

| No. | Cohort | T2D | Control |
| --- | --- | --- | --- |
| 1 | BioMe™ BioBank Program (BioMe) | 252 | 1,590 |
| 2 | deCODE genetics (deCODE) | 11,448 | 278,375 |
| 3 | Diabetes Gene Discovery Group (DGDG) | 677 | 697 |
| 4 | Diabetes Genetics (DGI) | 1,021 | 1,063 |
| 5 | Estonian Genome Center, University of Tartu (EGCUT_ExomeCore) | 79 | 4,507 |
| 6 | Estonian Genome Center, University of Tartu (EGCUT_Human370CNV) | 150 | 1,893 |
| 7 | Estonian Genome Center, University of Tartu (EGCUT_OmniExpress) | 673 | 6,646 |
| 8 | Framingham Heart Study (FHS) | 1,138 | 7,153 |
| 9 | Finland-United States Investigation of NIDDM Genetics (FUSION) | 1,045 | 1,164 |
| 10 | German Chronic Kidney Disease (GCKD) | 1,778 | 3,256 |
| 11 | Genetic Epidemiology Network of Arteriosclerosis (GENOA) | 144 | 1,116 |
| 12 | Resource for Genetic Epidemiology on Adult Health and Aging (GERA) | 6,961 | 13,922 |
| 13 | Genetics of Diabetes and Audit Research in Tayside Scotland (GoDARTS) | 2,993 | 2,641 |
| 14 | GoMAP (Genetic Overlap between Metabolic and Psychiatric traits) & TEENAGE (TEENs of Attica: Genes and Environment) (GoMAP-TEENAGE) | 412 | 390 |
| 15 | Health Professional Follow-Up Study (HPFS) | 1,094 | 1,259 |
| 16 | InterAct Consortium (INTERACT_coreexome) | 5,125 | 7,275 |
| 17 | InterAct Consortium (INTERACT_GWAS) | 4,190 | 4,257 |
| 18 | KORAgen Study Helmholtz zentrum München (KORA) | 1,084 | 2,852 |
| 19 | Multi-Ethnic Study of Atherosclerosis (MESA) | 174 | 2,493 |
| 20 | METabolic Syndrome in Men (METSIM) | 1,350 | 5,178 |
| 21 | Michigan Genomics Initiative (MGI) | 1,987 | 14,906 |
| 22 | Nurses' Health Study (NHS) | 1,453 | 1,731 |
| 23 | NUgene Project (NUGENE) | 521 | 600 |
| 24 | Prospective Investigation of the Vasculature in Uppsala Seniors (PIVUS) | 111 | 838 |
| 25 | PROspective Study of Pravastatin in the Elderly at Risk (PROSPER) | 802 | 4,385 |
| 26 | Rotterdam Study (RS1) | 477 | 3,051 |
| 27 | Rotterdam Study (RS2) | 275 | 1,877 |
| 28 | Rotterdam Study (RS3) | 281 | 2,746 |
| 29 | UK BioBank | 19,119 | 423,698 |
| 30 | Uppsala Longitudinal Study of Adult Men (ULSAM) | 166 | 953 |
| 31 | Danish T2D case-control study (UPCH) | 5,220 | 18,556 |
| 32 | Wellcome Trust Case-Control Consortium (WTCCC) | 1,924 | 2,938 |

Samples are genotyped with a variety of genome-wide SNP arrays. Array quality control criteria implemented within each study by excluding variants with: (i) allele frequencies differed from those for European ancestry haplotypes from the HRC reference panel (5) by more than 20%; (ii)AT/GC variants had MAF>40% because of potential undetected errors in strand alignment; or (iii) MAF<1% because of difficulties in calling rare variants. Each genotype scaffold for each study, with exception of the deCODE GWAS, was then imputed up to the HRC reference panel. The GWAS from deCODE was imputed up to a reference panel based on 30,440 Icelandic whole-genome sequences (6), and only variants that were present on the HRC panel were considered for downstream analyses.

The quality of the GWAS datasets was controlled by applying the following data filters: variants with INFO ≥0.80 if they existed were filtered in; variants with consistent alleles among each dataset were checked to adjust two situations: palindromic alleles and opposite alleles. In total, 8, 335, 938 variants for SCZ_EAS and 9, 745, 488 for SCZ_EUR, 11, 825, 585 for T2D_EAS and 13, 583, 104 for T2D_EUR were considered for the next analysis.

**1.2LD (linkage disequilibrium) score regression**

As that variants in high LD have higher test statistics on average than variants within low LD and expected magnitude of statistical inflation caused by genetic drift or sample overlap does not correlate with LD score, LD score regression is developed to estimate genetic correlation from summary GWAS statistics. Here, through using LDSC (v1.0.1) cross-trait LD score regression analysis, which is a simple extension of general single-trait LD score regression, was performed to analyze the genetic correlation between SCZ and T2D (7, 8). Firstly, GWAS summary statistics were reformatted, and SNPs were filtered by 1.1 million variants, subset of 1000 Genomes and HapMap3(9) with MAF below 0.05 and long-range LD MHC regions. Secondly, the pre-computed LD scores for each population (downloaded from <https://data.broadinstitute.org/alkesgroup/LDSCORE/>), which were built on the 1000 Genomes project phase 3 (https://www.internationalgenome.org), were used as reference to estimate the LD Score regression intercept, heritability with each disorder, and then to estimate the observed scale genetic correlation with two disorders since T2D has varied population prevalence (10). To compare the significance of the difference between the genetic correlations between of EAS and EUR population, Fisher’s Z-score transformed from rg using the following formula(11):

$Z_{r_{g}}=\frac{1}{2}ln(\frac{1+r_{g}}{1-r_{g}})$ and $Z_{r12}=\frac{|Z_{r1}-Z_{r2}|}{s}$

Here, r_g_, r1 and r2 are represented as the genetic correlation per trait, s is calculated with the following formulat:

s=$\sqrt{\frac{1}{n_{r1}-3}+\frac{1}{n_{r2}-3}}$

Here, nr1 and nr2 are represented as the effect sample size per correlation, and calculated based on:

$$n_{r_{g}}=2+\frac{1-r_{g}}{{se}^{2}}$$

Here, se is represented as the standard error for the genetic correlation. The two-tailed P value was calculated from the Z-score of a standard normal distribution.

Moreover, partitioned LDSC analysis was performed to estimate the genetic correlation of these two diseases for each autosome.

**1.3 Local genetic correlation analysis**

To investigate the local independent genomic region of SCZ shared genetic overlap with T2D, Heritability Estimation from Summary Statistics software package (HESS, v0.5.3-beta) (12) was implemented to evaluate the local genetic correlations. There are totally three steps, i.e. S1, computing eigenvalues, squared projections, and product of projections; S2, estimating local SNP-heritability of each trait; S3, estimating local genetic covariance and standard error. A total of 1443 and 1702 approximately independent LD blocks built on 1000 Genomes phase 3 for EAS and EUR respectively were checked as genome partition loci by HESS(13). For each population, the independent blocks include five regions in the MHC, for example, chromosome 6: 28,017,819–28,917,608, 28,917,608–29,737,971, 30,798,168–31,571,218, 31,571,218–32,682,664, and 32,682,664–33,236,497 for EUR population. The local genetic correlation was calculated from the local single-trait SNP heritability and local cross-trait genetic covariance estimates with following formula:

$$r_{L}=\frac{{cov}_{L}}{\sqrt{{h_{L}^{2}(SCZ)h_{L}^{2} (T2D)}}}$$

Here, cov_L_ was the local genetic covariance obtained from the third step of HESS, $h_{L}^{2}$(SCZ) and $h_{L}^{2}$(T2D) were the estimated local heritability of each disease obtained from the second step.

**1.4 Polygenic overlap analysis**

Regardless of the genetic correlation between two traits, MiXeR (v1.3) was utilized to qualify the polygenic overlap by estimating the total number of shared and trait-specific causal variants between two diseases and calculating the proportion of these shared causal variants in the total number of causal variants considered, and present the result in form of Venn diagram (14). Based on the causal mixture model with Gaussian distribution, MiXeR assumes that only a small fraction of variants has an effect on the trait, while the remaining variants do not. To avoid taking infinitesimally small effects, the presented numbers of causal variants only took over 22.6% of their total estimate and jointly accounted for 90% of SNP heritability in each disease(14). And the effects of the LD structure, the minor allele frequency (MAF), and the sample size were accounted for estimating the total number of causal variants. As a direct extension of cross-trait LD score regression by relaxing the infinitesimal assumption, MiXeR took 9,997,231 SNPs from 1000 Genomes Phase 3 data were used to estimate the LD structure.

**1.5 Mendelian randomization (MR) analysis**

Based on that genetic variants are associated with the exposure factor; not related to confounding factors associated with the outcome; and must affect the outcome through exposure factors, MR method is developed to infer the causal relation between the exposure factors and outcome (15). Genetic variants from GWAS usually are treated as instrumental variants for their stability and randomness, however they may cause horizontal pleiotropy that variants affect outcome and exposure traits via a shared heritable factor (correlated pleiotropy) or separate mechanisms (uncorrelated pleiotropy) other than a causal effect (16). Here, multiple MR methods with different assumptions on the extent and nature of horizontal pleiotropy were utilized to obtain a reliable and noteworthy results.

Genome-wide Complex Trait Analysis tool (GCTA v1.93.3beta2) was firstly used to analyze the bidirectional causal links between SCZ and T2D based on Generalized Summary-data-based Mendelian Randomization (GSMR) analysis (17). Let y be the liability of a disease on the logit scale, x be a exposure factor in standard deviation (SD) units and z be the genotype of a SNP. The MR estimate of the causal effect of exposure factor on disease is:
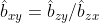
 , where
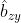
 is the effect of z on y on the logit scale of odds ratio,
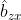
 is the effect of z on x, and
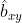
 is the effect of x on y free of confounding from non-genetic factors. GSMR can integrate the
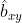
 estimates of all the non-pleiotropic SNP by Generalized Least Squares (GLS) through taking the
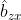
 and
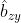
 for each SNP and the linkage disequilibrium (LD) between SNPs comprehensively in account. Furthermore, the estimate of
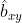
 by GSMR is unbiased under the alternative hypothesis that
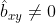
 and that
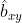
 approximately equals to logOR (17). Here, GSMR utilizes the following parameters: P≤5×10^-8^ as the GWAS threshold to select variants for independence analysis, r2 =0.05 as the LD threshold to identify independent SNP based on 1000 genome project (phase3) population reference, P=0.01 as the threshold for HEIDI-outlier analysis approach to detect SNPs with horizontal pleiotropic effects and remove them(18), and 10 as the minimum number of instrumental SNPs required for MR analysis.

Furthermore, the inverse variance weighting (IVW)(19), maximum likelihood (ML)(20), weighted median(21), were implemented to explore putative causal relationships between SCZ and T2D through using R packages of TwoSampleMR(16). IVW assumes no uncorrelated pleiotropy, to which MR-Egger may add an intercept to exclude confounding from such pleiotropy. The ML assumes the valid instrumental variables form the largest group of SNPs among all the groups may give different ratio estimates, thus may control type I errors with high power. While the weighted median capable of accounting for some correlated pleiotropy can identify true causality if ≤50% of the weights are from invalid SNPs by measuring the weighted median rather than weighted mean of the SNP ratio, which. For these three MR methods, independent SNPs (LD clumping r2 ≤ 0.05 within 1000-kb based on the 1000 genome European reference) with evidence for genome-wide association (p ≤ 5 × 10−8) with the ‘exposure’ disorder were used as instrumental variables, and merged with the SNPs from the ‘outcome’ disorder or trait. And the MR-Egger regression and MR-PRESSO models were used to determine the directional pleiotropy through using R packages of MR-eggr and MRPRESSO (22, 23).

**1.6 Genome-wide cross-trait/ethnic meta-analysis**

The Cross Phenotype Association (CPASSOC) method (24) was employed to identify shared variants between SCZ and T2D, which allows for the presence of heterogeneous effects across traits and provides statistic SHet and P value weighted by a sample size. During the CPASSOC analysis, there were two steps: one was to estimate the correlation matrix with SNPs whose summary statistics Z scores were greater than 1.96 or less than −1.96 and which had linkage disequilibrium (LD) pattern from 1000 genome projects phase3, the other was to perform S_Hom_ and S_Het_ tests. S_Hom_ is more powerful when heterogeneity is not present, while S_Het_ allows for trait heterogeneity effects. The significant level P = 5 × 10^−8^ was applied as in GWAS.

**1.7 Positional gene mapping within the platform FUMA**

Moreover, to map and prioritize genes MAGMA gene identification analysis within the platform FUMA was performed with the SNP-wide mean model under the 1000 Genome Phase3 population reference (25). During the analysis, genes within 100 kb of each candidate SNPs were mapped and prioritized, which are in LD with genome-wide significant SNPs at the adjusted r2 threshold.

**1.8 MAGMA gene property analysis for tissue specificity and Cell type-specificity**

Furthermore, based on the regression model with SNPs MAGMA gene property analysis for tissue specificity was also performed to test relationships between tissue specific gene expression profiles and disease-gene associations, i.e. to identify tissue specificity of the cross-trait significant SNPs, through using the FUMA platform that is a web tool of Functional Mapping and Annotation of GWAS (26).

In order to determine the effective cell type in Human peripheral blood mononuclear cells (PBMCs) for both SCZ and T2D, 10x Genomics’ single-cell RNA-seq (scRNA-seq) were extracted(27). MAGMA gene-property analysis was performed to test cell type specificity of phenotype with GWAS summary statistics using the FUMA platform(26).

**References:**

1. Lam M, Chen CY, Li Z, Martin AR, Bryois J, Ma X, et al. Comparative genetic architectures of schizophrenia in East Asian and European populations. Nature genetics. 2019;51(12):1670-8. doi: 10.1038/s41588-019-0512-x. PubMed PMID: 31740837; PubMed Central PMCID: PMC6885121.

2. Schizophrenia Working Group of the Psychiatric Genomics C. Biological insights from 108 schizophrenia-associated genetic loci. Nature. 2014;511(7510):421-7. doi: 10.1038/nature13595. PubMed PMID: 25056061; PubMed Central PMCID: PMC4112379.

3. Spracklen CN, Horikoshi M, Kim YJ, Lin K, Bragg F, Moon S, et al. Identification of type 2 diabetes loci in 433,540 East Asian individuals. Nature. 2020;582(7811):240-5. doi: 10.1038/s41586-020-2263-3. PubMed PMID: 32499647; PubMed Central PMCID: PMC7292783.

4. Mahajan A, Taliun D, Thurner M, Robertson NR, Torres JM, Rayner NW, et al. Fine-mapping type 2 diabetes loci to single-variant resolution using high-density imputation and islet-specific epigenome maps. Nat Genet. 2018;50(11):1505-13. Epub 2018/10/10. doi: 10.1038/s41588-018-0241-6. PubMed PMID: 30297969; PubMed Central PMCID: PMCPMC6287706.

5. McCarthy S, Das S, Kretzschmar W, Delaneau O, Wood AR, Teumer A, et al. A reference panel of 64,976 haplotypes for genotype imputation. Nature genetics. 2016;48(10):1279-83. doi: 10.1038/ng.3643. PubMed PMID: 27548312; PubMed Central PMCID: PMC5388176.

6. Jonsson H, Sulem P, Kehr B, Kristmundsdottir S, Zink F, Hjartarson E, et al. Whole genome characterization of sequence diversity of 15,220 Icelanders. Scientific data. 2017;4:170115. doi: 10.1038/sdata.2017.115. PubMed PMID: 28933420; PubMed Central PMCID: PMC5607473.

7. Bulik-Sullivan BK, Loh PR, Finucane HK, Ripke S, Yang J, Schizophrenia Working Group of the Psychiatric Genomics C, et al. LD Score regression distinguishes confounding from polygenicity in genome-wide association studies. Nature genetics. 2015;47(3):291-5. doi: 10.1038/ng.3211. PubMed PMID: 25642630; PubMed Central PMCID: PMC4495769.

8. Bulik-Sullivan B, Finucane HK, Anttila V, Gusev A, Day FR, Loh PR, et al. An atlas of genetic correlations across human diseases and traits. Nature genetics. 2015;47(11):1236-41. doi: 10.1038/ng.3406. PubMed PMID: 26414676; PubMed Central PMCID: PMC4797329.

9. International HapMap C, Altshuler DM, Gibbs RA, Peltonen L, Altshuler DM, Gibbs RA, et al. Integrating common and rare genetic variation in diverse human populations. Nature. 2010;467(7311):52-8. doi: 10.1038/nature09298. PubMed PMID: 20811451; PubMed Central PMCID: PMC3173859.

10. Genomes Project C, Auton A, Brooks LD, Durbin RM, Garrison EP, Kang HM, et al. A global reference for human genetic variation. Nature. 2015;526(7571):68-74. doi: 10.1038/nature15393. PubMed PMID: 26432245; PubMed Central PMCID: PMC4750478.

11. Yang Y, Musco H, Simpson-Yap S, Zhu Z, Wang Y, Lin X, et al. Investigating the shared genetic architecture between multiple sclerosis and inflammatory bowel diseases. Nature communications. 2021;12(1):5641. doi: 10.1038/s41467-021-25768-0. PubMed PMID: 34561436; PubMed Central PMCID: PMC8463615.

12. Shi H, Kichaev G, Pasaniuc B. Contrasting the Genetic Architecture of 30 Complex Traits from Summary Association Data. American journal of human genetics. 2016;99(1):139-53. doi: 10.1016/j.ajhg.2016.05.013. PubMed PMID: 27346688; PubMed Central PMCID: PMC5005444.

13. Berisa T, Pickrell JK. Approximately independent linkage disequilibrium blocks in human populations. Bioinformatics. 2016;32(2):283-5. doi: 10.1093/bioinformatics/btv546. PubMed PMID: 26395773; PubMed Central PMCID: PMC4731402.

14. Frei O, Holland D, Smeland OB, Shadrin AA, Fan CC, Maeland S, et al. Bivariate causal mixture model quantifies polygenic overlap between complex traits beyond genetic correlation. Nature communications. 2019;10(1):2417. doi: 10.1038/s41467-019-10310-0. PubMed PMID: 31160569; PubMed Central PMCID: PMC6547727.

15. Pierce BL, Burgess S. Efficient design for Mendelian randomization studies: subsample and 2-sample instrumental variable estimators. American journal of epidemiology. 2013;178(7):1177-84. doi: 10.1093/aje/kwt084. PubMed PMID: 23863760; PubMed Central PMCID: PMC3783091.

16. Smith GD, Ebrahim S. 'Mendelian randomization': can genetic epidemiology contribute to understanding environmental determinants of disease? Int J Epidemiol. 2003;32(1):1-22. Epub 2003/04/12. doi: 10.1093/ije/dyg070. PubMed PMID: 12689998.

17. Zhu Z, Zheng Z, Zhang F, Wu Y, Trzaskowski M, Maier R, et al. Causal associations between risk factors and common diseases inferred from GWAS summary data. Nature communications. 2018;9(1):224. doi: 10.1038/s41467-017-02317-2. PubMed PMID: 29335400; PubMed Central PMCID: PMC5768719.

18. Zhu Z, Zhang F, Hu H, Bakshi A, Robinson MR, Powell JE, et al. Integration of summary data from GWAS and eQTL studies predicts complex trait gene targets. Nature genetics. 2016;48(5):481-7. doi: 10.1038/ng.3538. PubMed PMID: 27019110.

19. Burgess S, Butterworth A, Thompson SG. Mendelian randomization analysis with multiple genetic variants using summarized data. Genetic epidemiology. 2013;37(7):658-65. doi: 10.1002/gepi.21758. PubMed PMID: 24114802; PubMed Central PMCID: PMC4377079.

20. Xue H, Shen X, Pan W. Constrained maximum likelihood-based Mendelian randomization robust to both correlated and uncorrelated pleiotropic effects. American journal of human genetics. 2021;108(7):1251-69. doi: 10.1016/j.ajhg.2021.05.014. PubMed PMID: 34214446; PubMed Central PMCID: PMC8322939.

21. Bowden J, Davey Smith G, Haycock PC, Burgess S. Consistent Estimation in Mendelian Randomization with Some Invalid Instruments Using a Weighted Median Estimator. Genetic epidemiology. 2016;40(4):304-14. doi: 10.1002/gepi.21965. PubMed PMID: 27061298; PubMed Central PMCID: PMC4849733.

22. Bowden J, Davey Smith G, Burgess S. Mendelian randomization with invalid instruments: effect estimation and bias detection through Egger regression. International journal of epidemiology. 2015;44(2):512-25. doi: 10.1093/ije/dyv080. PubMed PMID: 26050253; PubMed Central PMCID: PMC4469799.

23. Verbanck M, Chen CY, Neale B, Do R. Detection of widespread horizontal pleiotropy in causal relationships inferred from Mendelian randomization between complex traits and diseases. Nature genetics. 2018;50(5):693-8. doi: 10.1038/s41588-018-0099-7. PubMed PMID: 29686387; PubMed Central PMCID: PMC6083837.

24. Zhu X, Feng T, Tayo BO, Liang J, Young JH, Franceschini N, et al. Meta-analysis of correlated traits via summary statistics from GWASs with an application in hypertension. American journal of human genetics. 2015;96(1):21-36. doi: 10.1016/j.ajhg.2014.11.011. PubMed PMID: 25500260; PubMed Central PMCID: PMC4289691.

25. de Leeuw CA, Mooij JM, Heskes T, Posthuma D. MAGMA: generalized gene-set analysis of GWAS data. PLoS computational biology. 2015;11(4):e1004219. doi: 10.1371/journal.pcbi.1004219. PubMed PMID: 25885710; PubMed Central PMCID: PMC4401657.

26. Watanabe K, Taskesen E, van Bochoven A, Posthuma D. Functional mapping and annotation of genetic associations with FUMA. Nature communications. 2017;8(1):1826. doi: 10.1038/s41467-017-01261-5. PubMed PMID: 29184056; PubMed Central PMCID: PMC5705698.

27. Zheng GX, Terry JM, Belgrader P, Ryvkin P, Bent ZW, Wilson R, et al. Massively parallel digital transcriptional profiling of single cells. Nature communications. 2017;8:14049. doi: 10.1038/ncomms14049. PubMed PMID: 28091601; PubMed Central PMCID: PMC5241818 L.M., D.A.M., S.Y.N., M.S.L., P.W.W., C.M.H., R.B., A.W., K.D.N., T.S.M. and B.J.H. are employees of 10x Genomics.

**Figure S1.** Flowchart of genomic analyses performed in the current study. SCZ, schizophrenia; T2D, type 2 diabetes.

**
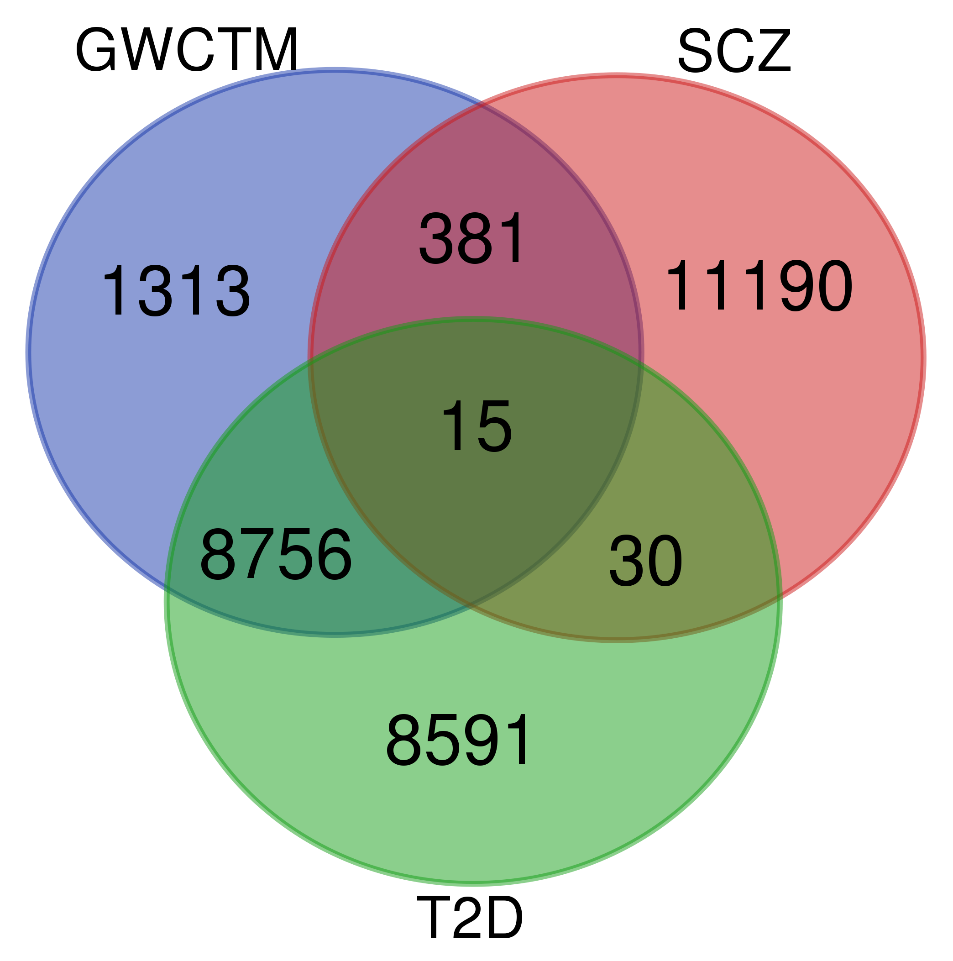
**

**Figure S2.** Venn diagrams of genome-wide significant SNPs identified with both GWCTM methods and those identified in either GWAS of SCZ or T2D

**
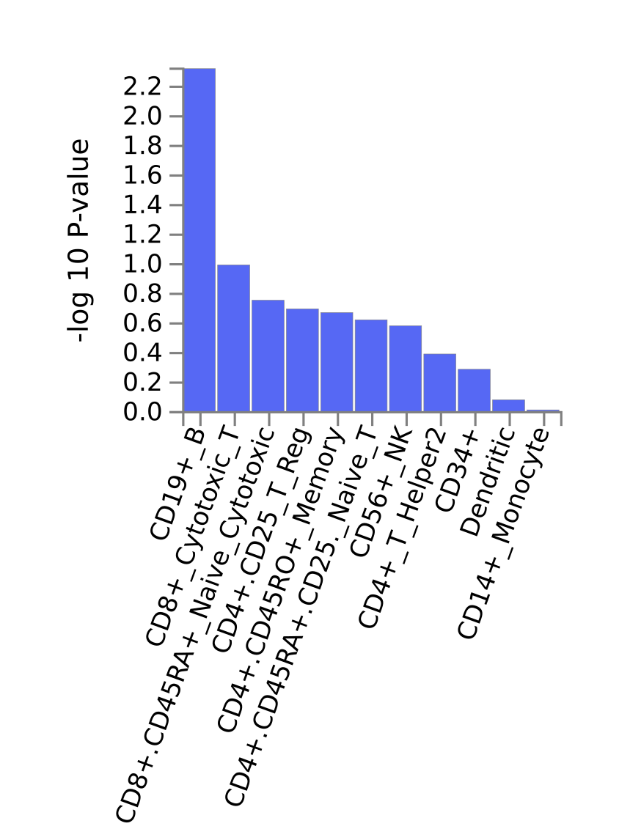
**

**Figure S3.** Cell type specific analysis with MAGMA

| Table S1. Partitioned genetic correlation in term of autosomes estimated through using LDSC method | | | | | | | |
| --- | --- | --- | --- | --- | --- | --- | --- |
| No constrained intercept | | | |  | Constrained intercept | | |
| CHR | rg | SE | P |  | rg | SE | P |
|  | EAS population | |  |  |  |  |  |
| 1 | -0.046 | 0.063 | 0.4645 |  | -0.1288 | 0.0905 | 0.1546 |
| 2 | -0.0755 | 0.0666 | 0.2571 |  | -0.1723 | 0.0906 | 0.0571 |
| 3 | -0.0861 | 0.0597 | 0.149 |  | -0.1099 | 0.0715 | 0.1244 |
| 4 | 0.0861 | 0.0807 | 0.2858 |  | 0.0464 | 0.1058 | 0.6613 |
| 5 | -0.005 | 0.0638 | 0.9375 |  | -0.032 | 0.0816 | 0.695 |
| 6 | -0.031 | 0.0588 | 0.5985 |  | 0.0291 | 0.0662 | 0.6608 |
| 7 | 0.1468 | 0.0626 | 0.019 |  | 0.087 | 0.0869 | 0.3169 |
| 8 | -0.0442 | 0.0608 | 0.4674 |  | -0.0011 | 0.0869 | 0.9903 |
| 9 | 0.0687 | 0.0818 | 0.4005 |  | 0.101 | 0.1777 | 0.5698 |
| 10 | -0.1465 | 0.0641 | 0.0224 |  | -0.1965 | 0.0933 | 0.0351 |
| 11 | -0.0847 | 0.0683 | 0.215 |  | -0.5344 | 0.3053 | 0.0801 |
| 12 | -0.0803 | 0.0759 | 0.2902 |  | -0.226 | 0.1122 | 0.0439 |
| 13 | 0.0855 | 0.1002 | 0.3936 |  | -0.0442 | 0.1581 | 0.7797 |
| 14 | 0.0635 | 0.0806 | 0.4308 |  | 0.0444 | 0.1166 | 0.7037 |
| 15 | -0.1587 | 0.1014 | 0.1177 |  | -0.1586 | 0.1412 | 0.2614 |
| 16 | 0.0194 | 0.0815 | 0.812 |  | 0.0726 | 0.1234 | 0.5559 |
| 17 | 0.0292 | 0.095 | 0.7589 |  | 0.1299 | 0.1728 | 0.4521 |
| 18 | 0.1688 | 0.1217 | 0.1653 |  | 0.1377 | 0.1541 | 0.3717 |
| 19 | -0.0425 | 0.1006 | 0.6724 |  | 0.1438 | 0.166 | 0.3864 |
| 20 | -0.0762 | 0.1038 | 0.4626 |  | -0.1503 | 0.1593 | 0.3454 |
| 21 | 0.191 | 0.2651 | 0.4711 |  | 0.5857 | 0.5903 | 0.3211 |
| 22 | -0.3036 | 0.1542 | 0.0489 |  | -0.5111 | 0.2826 | 0.0705 |
|  | EUR population | |  |  |  |  |  |
| 1 | -0.1605 | 0.0487 | 0.001 |  | -0.2207 | 0.077 | 0.0042 |
| 2 | -0.0695 | 0.0445 | 0.1179 |  | -0.0566 | 0.0761 | 0.4571 |
| 3 | -0.0818 | 0.0549 | 0.1365 |  | -0.2533 | 0.0861 | 0.0033 |
| 4 | -0.073 | 0.0595 | 0.2203 |  | -0.1942 | 0.1069 | 0.0693 |
| 5 | -0.0891 | 0.0593 | 0.1328 |  | -0.1504 | 0.103 | 0.1444 |
| 6 | -0.0135 | 0.0541 | 0.8027 |  | -0.138 | 0.1057 | 0.1916 |
| 7 | -0.0107 | 0.0474 | 0.822 |  | -0.027 | 0.0907 | 0.7662 |
| 8 | -0.0031 | 0.065 | 0.9621 |  | 0.0328 | 0.1034 | 0.7509 |
| 9 | -0.0669 | 0.0634 | 0.2918 |  | -0.2805 | 0.1861 | 0.1318 |
| 10 | -0.0694 | 0.0535 | 0.1947 |  | 0.0763 | 0.1118 | 0.4953 |
| 11 | -0.0955 | 0.0647 | 0.14 |  | -0.1887 | 0.1498 | 0.2079 |
| 12 | -0.0121 | 0.0665 | 0.8558 |  | -0.0673 | 0.1465 | 0.6459 |
| 13 | -0.1948 | 0.0795 | 0.0143 |  | -0.3086 | 0.1491 | 0.0385 |
| 14 | -0.1062 | 0.0878 | 0.2265 |  | -0.1365 | 0.1621 | 0.3996 |
| 15 | -0.1244 | 0.0653 | 0.0568 |  | 0.0371 | 0.099 | 0.7078 |
| 16 | -0.0964 | 0.0609 | 0.1135 |  | -0.104 | 0.0937 | 0.2672 |
| 17 | -0.0422 | 0.0803 | 0.5991 |  | 0.0217 | 0.1478 | 0.8831 |
| 18 | -0.0237 | 0.0856 | 0.7814 |  | 0.0687 | 0.1046 | 0.5113 |
| 19 | 0.0337 | 0.0819 | 0.6808 |  | 0.0591 | 0.132 | 0.6545 |
| 20 | -0.0884 | 0.0808 | 0.2744 |  | -0.0335 | 0.1485 | 0.8214 |
| 21 | -0.3206 | 0.1456 | 0.0276 |  | -0.7477 | 0.4163 | 0.0725 |
| 22 | 0.0166 | 0.0879 | 0.8506 |  | 0.0314 | 0.1029 | 0.7605 |

| Table S2. Local genetic correlation estimated through using HESS method | | | | |
| --- | --- | --- | --- | --- |
| Loci | Nsnp | rL | SE | P |
| EAS population |  |  |  |  |
| chr1:3492739-4543012 | 2531 | -0.000275 | 0.000114 | 0.01605 |
| chr1:39550621-41288115 | 2463 | -0.000238 | 0.000121 | 0.049599 |
| chr2:20402938-23703344 | 4357 | 0.0002404 | 0.000116 | 0.037438 |
| chr2:40508070-42336744 | 3736 | -0.000252 | 0.000112 | 0.024473 |
| chr2:224859323-227665678 | 4523 | -0.000286 | 0.00012 | 0.017233 |
| chr6:118099644-120338588 | 3672 | -0.00024 | 0.000111 | 0.030434 |
| chr7:1240747-2482290 | 2496 | -0.000326 | 0.000124 | 0.008515 |
| chr7:26552261-28554104 | 2804 | 0.000256 | 0.00012 | 0.033373 |
| chr7:126869221-129589868 | 3515 | 0.0005373 | 0.000157 | 0.000626 |
| chr7:146397904-147996405 | 2860 | 0.0002234 | 0.000113 | 0.047079 |
| chr8:142610656-144214290 | 3597 | 0.0003119 | 0.000113 | 0.005788 |
| chr10:71054709-72673145 | 3471 | -0.000281 | 0.00013 | 0.031078 |
| chr10:89149284-90787308 | 2203 | -0.000232 | 0.000115 | 0.043527 |
| chr11:47003018-54695473 | 7047 | 0.0003516 | 0.000127 | 0.005701 |
| chr11:90954609-92731401 | 2787 | -0.000236 | 0.000119 | 0.047341 |
| chr11:121769481-123339958 | 2832 | -0.000229 | 0.000114 | 0.043468 |
| chr12:107548626-109270450 | 3115 | -0.00026 | 0.000122 | 0.033341 |
| chr14:63291421-65225452 | 3046 | -0.000247 | 0.000117 | 0.035205 |
| chr15:40384324-41798467 | 1841 | -0.000351 | 0.000139 | 0.01182 |
| chr15:51678516-53699684 | 3580 | -0.00027 | 0.000121 | 0.025953 |
| chr15:90070118-92011845 | 3453 | -0.00035 | 0.00014 | 0.01259 |
| chr17:45880185-49895548 | 5361 | 0.0002876 | 0.000118 | 0.014783 |
| chr19:45579043-47334409 | 3061 | 0.0005065 | 0.000148 | 0.000619 |
| EUR population |  |  |  |  |
| chr1:1892607-3582736 | 2597 | 0.0001145 | 5.33E-05 | 0.031609 |
| chr1:10806984-11777841 | 1296 | 0.0001119 | 4.82E-05 | 0.020233 |
| chr1:27401867-30161881 | 2881 | -0.000122 | 5.65E-05 | 0.030835 |
| chr1:38731847-40200567 | 2049 | -0.000139 | 5.43E-05 | 0.010618 |
| chr1:59890409-61922365 | 2434 | 0.0001388 | 5.39E-05 | 0.010033 |
| chr1:84844495-86454751 | 2836 | -0.000128 | 4.69E-05 | 0.006279 |
| chr1:102898745-103914211 | 1685 | -0.000137 | 4.46E-05 | 0.002139 |
| chr1:159913048-162346721 | 3787 | -0.000115 | 4.79E-05 | 0.016752 |
| chr1:169086324-170557776 | 3118 | -0.000141 | 4.87E-05 | 0.00373 |
| chr1:177433381-178944309 | 2135 | -0.000122 | 5.56E-05 | 0.02818 |
| chr1:194107442-196176201 | 4069 | -0.000101 | 5.14E-05 | 0.049427 |
| chr1:196176201-197311514 | 1211 | -8.38E-05 | 4.00E-05 | 0.035973 |
| chr1:216243634-218705513 | 4461 | 9.30E-05 | 4.59E-05 | 0.042507 |
| chr1:218705513-219590571 | 1716 | -0.000165 | 4.59E-05 | 0.000315 |
| chr1:240564091-241582220 | 1917 | -0.000152 | 4.60E-05 | 0.000941 |
| chr2:60292000-62429044 | 2646 | -0.000128 | 5.66E-05 | 0.023293 |
| chr2:73174848-75630086 | 3172 | -0.000149 | 5.92E-05 | 0.011641 |
| chr2:75630086-76913661 | 2458 | -9.82E-05 | 4.99E-05 | 0.049329 |
| chr2:85140680-88038507 | 3006 | -0.000215 | 5.13E-05 | 2.72E-05 |
| chr2:103588215-105125034 | 1584 | 0.0001084 | 5.15E-05 | 0.035431 |
| chr2:116772470-118367466 | 2539 | 8.99E-05 | 4.37E-05 | 0.039908 |
| chr2:158533218-159577082 | 1756 | -9.70E-05 | 4.92E-05 | 0.04868 |
| chr2:159577082-161769733 | 3755 | 0.0001159 | 5.34E-05 | 0.029968 |
| chr2:196447456-198078110 | 2055 | -0.000137 | 5.74E-05 | 0.016866 |
| chr3:8648561-9543183 | 1680 | 9.17E-05 | 4.67E-05 | 0.049579 |
| chr3:13070799-14816900 | 3590 | 0.0001398 | 5.08E-05 | 0.005904 |
| chr3:20091348-21643707 | 3536 | -9.11E-05 | 4.30E-05 | 0.034173 |
| chr3:30717955-32351715 | 2854 | -0.000153 | 5.37E-05 | 0.004507 |
| chr3:32351715-33255592 | 1580 | -0.000105 | 4.55E-05 | 0.020434 |
| chr3:33255592-35283458 | 3137 | -0.000116 | 5.87E-05 | 0.047275 |
| chr3:36486842-38356116 | 2376 | -0.000124 | 5.96E-05 | 0.037063 |
| chr3:46657500-47727212 | 856 | -8.49E-05 | 4.13E-05 | 0.039747 |
| chr3:69276762-70449028 | 2125 | 0.0001156 | 5.23E-05 | 0.027212 |
| chr3:70449028-72529329 | 3108 | 0.0001163 | 5.62E-05 | 0.038596 |
| chr3:84367479-85582231 | 2090 | -9.33E-05 | 4.72E-05 | 0.048163 |
| chr3:128194861-130244735 | 2764 | 0.0001066 | 4.70E-05 | 0.02352 |
| chr3:143164628-144410816 | 1860 | -0.000123 | 4.89E-05 | 0.011613 |
| chr3:151348730-153256571 | 3257 | -0.000116 | 5.63E-05 | 0.039925 |
| chr3:167117429-168580960 | 1895 | -0.00013 | 5.63E-05 | 0.021294 |
| chr3:179374586-181511166 | 2461 | -0.000124 | 6.22E-05 | 0.046827 |
| chr3:194059671-194786674 | 2137 | -9.53E-05 | 4.38E-05 | 0.029386 |
| chr4:1478711-2842979 | 2088 | 0.0001065 | 4.84E-05 | 0.027651 |
| chr4:8152235-9326479 | 1232 | 0.0001095 | 4.53E-05 | 0.015576 |
| chr4:15147446-15927009 | 1271 | 0.0001268 | 4.14E-05 | 0.002166 |
| chr4:15927009-17383322 | 2790 | 0.0001212 | 4.68E-05 | 0.009584 |
| chr4:74592390-77130707 | 4111 | -0.000138 | 5.25E-05 | 0.008583 |
| chr4:83372593-84799656 | 2509 | 0.0001362 | 4.90E-05 | 0.00546 |
| chr4:122657987-124286481 | 2250 | -0.000123 | 5.00E-05 | 0.014155 |
| chr4:158743718-161058864 | 3253 | -0.000105 | 5.08E-05 | 0.039249 |
| chr5:22152073-23494324 | 2192 | 0.0001018 | 5.09E-05 | 0.045723 |
| chr5:57340073-58524622 | 2624 | -0.000102 | 5.03E-05 | 0.043496 |
| chr5:87389991-88891530 | 1237 | 0.0001283 | 6.30E-05 | 0.041781 |
| chr5:115831047-117346213 | 2893 | -0.000109 | 5.01E-05 | 0.029434 |
| chr5:140645971-142981248 | 3416 | -0.000136 | 4.97E-05 | 0.006314 |
| chr5:148662624-150561298 | 3130 | -9.33E-05 | 4.55E-05 | 0.040346 |
| chr5:150561298-152867774 | 4192 | 0.0001557 | 6.24E-05 | 0.012606 |
| chr5:152867774-153773088 | 1869 | -0.000179 | 5.58E-05 | 0.001366 |
| chr5:165642395-166847740 | 1877 | -0.000102 | 4.75E-05 | 0.030952 |
| chr6:5791472-6785207 | 2264 | 0.0001026 | 4.34E-05 | 0.018041 |
| chr6:19207487-21684065 | 4515 | -0.000184 | 6.29E-05 | 0.003523 |
| chr6:25684587-26791233 | 2040 | -0.000171 | 7.45E-05 | 0.021542 |
| chr6:55468270-56105313 | 1410 | 8.25E-05 | 3.97E-05 | 0.03784 |
| chr6:61880512-63552888 | 3127 | 0.0001622 | 4.54E-05 | 0.000349 |
| chr6:63552888-65765742 | 3373 | 0.0002746 | 5.98E-05 | 4.36E-06 |
| chr6:93428644-94118142 | 1572 | -0.000127 | 5.26E-05 | 0.015645 |
| chr6:97093511-97842284 | 1185 | 0.0001351 | 4.52E-05 | 0.002817 |
| chr6:108464380-110304247 | 2442 | -0.000151 | 5.12E-05 | 0.003262 |
| chr6:110304247-112345014 | 2981 | -0.000127 | 5.41E-05 | 0.019309 |
| chr6:123856181-125424383 | 2295 | 0.0001357 | 5.71E-05 | 0.017489 |
| chr6:139845436-142288479 | 3078 | -0.000126 | 4.90E-05 | 0.010375 |
| chr7:12635461-13884202 | 3159 | 0.0001005 | 4.84E-05 | 0.037854 |
| chr7:44763828-45952922 | 2072 | 0.0001235 | 4.79E-05 | 0.009894 |
| chr7:71874885-73334602 | 1231 | -0.000101 | 5.02E-05 | 0.044101 |
| chr7:97099570-98715474 | 2631 | -9.78E-05 | 4.81E-05 | 0.042275 |
| chr8:3783017-4480476 | 2883 | 0.0001312 | 5.10E-05 | 0.010144 |
| chr8:4480476-5146927 | 2336 | 0.0001159 | 5.33E-05 | 0.02989 |
| chr8:11278998-13491775 | 4349 | 0.000212 | 5.77E-05 | 0.000238 |
| chr8:20060856-21661737 | 3260 | 0.0001111 | 4.61E-05 | 0.015954 |
| chr8:29327896-31133729 | 2608 | 9.56E-05 | 4.68E-05 | 0.041143 |
| chr8:31133729-31548360 | 826 | -9.92E-05 | 4.30E-05 | 0.020932 |
| chr8:111850847-113064320 | 1536 | -0.000103 | 5.06E-05 | 0.042821 |
| chr9:28811584-30387392 | 3557 | -0.00011 | 4.93E-05 | 0.026186 |
| chr9:36743283-38641599 | 3166 | -0.000145 | 5.41E-05 | 0.007282 |
| chr9:84211233-85440801 | 1371 | 0.0002229 | 6.21E-05 | 0.000329 |
| chr9:110695062-112778024 | 4411 | 0.0001018 | 4.93E-05 | 0.039123 |
| chr9:121321537-122260297 | 1391 | -0.000117 | 5.38E-05 | 0.029549 |
| chr9:122260297-124871322 | 3135 | 0.0002226 | 5.66E-05 | 8.47E-05 |
| chr10:12586797-13321600 | 1799 | -0.000109 | 4.50E-05 | 0.015421 |
| chr10:33707968-35109355 | 2142 | -0.000137 | 4.91E-05 | 0.005169 |
| chr10:43894771-44730075 | 1699 | 9.72E-05 | 4.62E-05 | 0.035314 |
| chr10:67549615-69900148 | 4820 | -0.000138 | 4.62E-05 | 0.002832 |
| chr10:73508512-75422550 | 1990 | 0.0001106 | 4.04E-05 | 0.006152 |
| chr10:78706814-80876749 | 3618 | 0.0001087 | 4.68E-05 | 0.020248 |
| chr10:100241302-100668400 | 639 | 8.07E-05 | 3.77E-05 | 0.03233 |
| chr11:15742552-17578402 | 2559 | -0.000232 | 5.95E-05 | 9.29E-05 |
| chr11:49866050-54695473 | 2690 | -9.60E-05 | 4.54E-05 | 0.034598 |
| chr11:70926292-72286017 | 1921 | 0.0001021 | 4.89E-05 | 0.036671 |
| chr11:114257728-114830666 | 979 | -6.82E-05 | 3.47E-05 | 0.049614 |
| chr11:116383348-117747110 | 2704 | -0.000133 | 4.79E-05 | 0.005448 |
| chr11:123500117-124495528 | 2306 | -0.000108 | 5.17E-05 | 0.036866 |
| chr11:126311320-127316679 | 2101 | 0.0001382 | 5.38E-05 | 0.010164 |
| chr11:127316679-128188537 | 1466 | -9.77E-05 | 4.39E-05 | 0.026049 |
| chr11:134205993-134946452 | 2172 | -0.000128 | 5.80E-05 | 0.026846 |
| chr12:4417679-5321472 | 1617 | 0.0001285 | 4.90E-05 | 0.008712 |
| chr12:55665837-57548860 | 1894 | 0.0001317 | 4.82E-05 | 0.006294 |
| chr12:65559695-67181144 | 2155 | 0.0001231 | 5.59E-05 | 0.027791 |
| chr12:70957987-72645075 | 2531 | -0.000178 | 5.44E-05 | 0.00106 |
| chr12:118135375-119754110 | 3134 | 0.0001036 | 5.16E-05 | 0.04472 |
| chr13:55817131-57554217 | 2202 | -0.00017 | 4.70E-05 | 0.000293 |
| chr13:57554217-58410626 | 1208 | -9.74E-05 | 4.15E-05 | 0.018896 |
| chr13:58410626-59302271 | 1444 | -0.000125 | 5.11E-05 | 0.014504 |
| chr13:61591949-63971559 | 4871 | -0.000123 | 5.17E-05 | 0.017889 |
| chr13:65200602-67844453 | 3798 | -0.000133 | 5.59E-05 | 0.017155 |
| chr13:75670143-77410555 | 3251 | 9.97E-05 | 4.55E-05 | 0.028484 |
| chr13:108189877-109121210 | 1855 | -0.000105 | 4.63E-05 | 0.023245 |
| chr13:112247592-113572488 | 2261 | 0.0001251 | 5.28E-05 | 0.017693 |
| chr14:41615502-43137389 | 2508 | 0.0001066 | 5.34E-05 | 0.045739 |
| chr14:57481262-59448336 | 3140 | 9.37E-05 | 4.68E-05 | 0.045369 |
| chr14:61680424-63790015 | 3534 | -0.000102 | 4.70E-05 | 0.030611 |
| chr14:71131957-72889615 | 2803 | -0.000213 | 6.63E-05 | 0.001338 |
| chr14:103012102-105001723 | 3030 | 0.0001803 | 6.10E-05 | 0.003094 |
| chr15:32441811-34015425 | 2589 | -9.17E-05 | 4.44E-05 | 0.039197 |
| chr15:50008043-51677560 | 2858 | -0.000123 | 5.23E-05 | 0.018622 |
| chr15:61265836-63215222 | 3348 | -0.000126 | 5.76E-05 | 0.029163 |
| chr15:76398624-78516053 | 3029 | -0.000116 | 5.76E-05 | 0.043379 |
| chr15:80860978-84260468 | 3704 | -0.000122 | 5.41E-05 | 0.024446 |
| chr15:84260468-86652905 | 4053 | -0.000138 | 5.51E-05 | 0.012063 |
| chr15:99244059-100636847 | 2924 | -0.000131 | 4.84E-05 | 0.006841 |
| chr16:2764829-4001196 | 1762 | 0.0001011 | 4.53E-05 | 0.025685 |
| chr16:14464002-16154060 | 1341 | -0.000103 | 4.75E-05 | 0.029989 |
| chr16:18643607-20150571 | 2219 | 0.0001169 | 5.16E-05 | 0.023522 |
| chr16:29036613-31382943 | 1715 | -0.000148 | 5.90E-05 | 0.012321 |
| chr16:31382943-46399473 | 779 | -8.06E-05 | 4.09E-05 | 0.048612 |
| chr16:63691589-65938566 | 3956 | -0.000115 | 5.77E-05 | 0.046754 |
| chr16:89041165-90292812 | 2354 | 0.0001342 | 5.44E-05 | 0.013623 |
| chr17:1172399-1928731 | 1511 | -0.000116 | 4.95E-05 | 0.018825 |
| chr17:16412342-18856320 | 2966 | 0.0001071 | 5.33E-05 | 0.04439 |
| chr17:34469036-36809344 | 2415 | -0.000183 | 5.35E-05 | 0.00061 |
| chr17:36809344-38877404 | 2246 | 0.0001552 | 5.86E-05 | 0.008114 |
| chr17:45876022-47517400 | 2605 | 0.0001608 | 5.27E-05 | 0.00229 |
| chr17:61545589-63148128 | 1387 | -0.000136 | 5.54E-05 | 0.014086 |
| chr18:1942911-3891658 | 4112 | -8.71E-05 | 4.35E-05 | 0.045316 |
| chr18:5834180-7090485 | 2225 | -0.000116 | 5.21E-05 | 0.025986 |
| chr18:24026191-25927682 | 2824 | -9.62E-05 | 4.58E-05 | 0.035416 |
| chr18:30264066-31780067 | 1707 | -0.000109 | 5.18E-05 | 0.035231 |
| chr18:47730584-51062185 | 5636 | 0.0001964 | 5.03E-05 | 9.41E-05 |
| chr18:51554175-55213838 | 4852 | -0.000281 | 5.91E-05 | 2.03E-06 |
| chr18:68621179-69621618 | 2356 | 0.0001194 | 4.54E-05 | 0.008545 |
| chr18:71971462-73526987 | 2513 | 0.0001364 | 5.35E-05 | 0.010776 |
| chr19:2098396-3019660 | 1736 | -0.000125 | 4.87E-05 | 0.010627 |
| chr19:3019660-4348967 | 1994 | 0.0001087 | 4.73E-05 | 0.021547 |
| chr19:4348967-5811852 | 2627 | 0.0001224 | 5.27E-05 | 0.020068 |
| chr20:8117011-9730921 | 2854 | -0.000111 | 4.27E-05 | 0.009445 |
| chr20:31614823-32813441 | 1169 | 0.0001497 | 5.21E-05 | 0.004099 |
| chr20:47199980-49239658 | 3613 | -0.000146 | 5.37E-05 | 0.006448 |
| chr21:15950982-18053165 | 2921 | -0.000115 | 4.93E-05 | 0.019209 |
| chr22:42690818-43714200 | 2143 | 0.0001669 | 5.51E-05 | 0.002445 |

| Table S3. 66 instrumental variables for the MR analysis of the causal effect of SCZ on T2D in EUR population | | | | | | | | |
| --- | --- | --- | --- | --- | --- | --- | --- | --- |
| SNP | CHR | BP | A1 | A2 | FRQ | BETA | SE | PVAL |
| rs1024582 | 12 | 2402246 | A | G | 0.357 | 0.099302 | 0.0114 | 2.71E-18 |
| rs1028885 | 6 | 25434518 | G | A | 0.218 | -0.0794 | 0.013 | 9.57E-10 |
| rs10749820 | 1 | 73812598 | C | G | 0.408 | 0.067098 | 0.011 | 1.26E-09 |
| rs1076884 | 16 | 13747803 | C | G | 0.245 | 0.076804 | 0.0127 | 1.55E-09 |
| rs10791097 | 11 | 130718630 | T | G | 0.477 | 0.076896 | 0.0109 | 1.61E-12 |
| rs11027857 | 11 | 24403620 | G | A | 0.475 | -0.0644 | 0.0109 | 2.95E-09 |
| rs11210892 | 1 | 44100084 | G | A | 0.342 | 0.067401 | 0.0115 | 4.13E-09 |
| rs11682175 | 2 | 57987593 | T | C | 0.512 | -0.0739 | 0.0112 | 4.61E-11 |
| rs117074560 | 6 | 96459651 | C | T | 0.9557 | 0.165795 | 0.0267 | 5.46E-10 |
| rs12163529 | 3 | 136298879 | G | A | 0.601 | 0.070498 | 0.011 | 1.59E-10 |
| rs1233578 | 6 | 28712247 | A | G | 0.877 | 0.188701 | 0.0161 | 1.48E-31 |
| rs12420205 | 11 | 113394035 | C | T | 0.295 | -0.0766 | 0.0119 | 1.39E-10 |
| rs12532143 | 7 | 111025948 | T | C | 0.669 | 0.080796 | 0.0115 | 2.33E-12 |
| rs12704290 | 7 | 86427626 | G | A | 0.886 | 0.105305 | 0.0168 | 3.48E-10 |
| rs13218591 | 6 | 26376832 | T | C | 0.726 | 0.092998 | 0.012 | 1.05E-14 |
| rs1610615 | 6 | 29711334 | C | T | 0.692 | 0.072495 | 0.0117 | 5.96E-10 |
| rs1615350 | 12 | 123650335 | C | T | 0.274 | 0.086103 | 0.0123 | 2.28E-12 |
| rs17194490 | 3 | 2547786 | G | T | 0.828 | -0.0992 | 0.0147 | 1.69E-11 |
| rs17531523 | 15 | 85132449 | C | G | 0.737 | 0.079098 | 0.0122 | 1.00E-10 |
| rs1805589 | 3 | 180647410 | C | T | 0.805 | 0.081004 | 0.0135 | 2.18E-09 |
| rs215412 | 4 | 23423586 | G | A | 0.656 | -0.0677 | 0.0115 | 3.56E-09 |
| rs217289 | 6 | 84401807 | G | A | 0.578 | 0.066396 | 0.011 | 1.58E-09 |
| rs2319280 | 1 | 150014017 | A | C | 0.153 | -0.0905 | 0.0148 | 9.61E-10 |
| rs2332700 | 14 | 72417326 | C | G | 0.261 | 0.077998 | 0.0125 | 4.27E-10 |
| rs2414718 | 15 | 61863133 | G | A | 0.401 | -0.0695 | 0.011 | 2.35E-10 |
| rs2428162 | 7 | 104595664 | T | C | 0.591 | -0.0663 | 0.0111 | 2.23E-09 |
| rs2802535 | 1 | 98508258 | C | T | 0.183 | -0.1174 | 0.0138 | 1.61E-17 |
| rs2810117 | 14 | 71391025 | T | C | 0.348 | 0.068602 | 0.0115 | 2.19E-09 |
| rs28681284 | 15 | 78908565 | C | T | 0.799 | 0.099102 | 0.0135 | 2.26E-13 |
| rs2949006 | 2 | 200715388 | T | G | 0.202 | 0.104 | 0.0137 | 3.45E-14 |
| rs3112532 | 5 | 152606570 | A | G | 0.331 | -0.0689 | 0.0114 | 1.57E-09 |
| rs34137090 | 8 | 111483782 | G | T | 0.786 | -0.0833 | 0.0133 | 4.45E-10 |
| rs3802924 | 11 | 133827733 | A | C | 0.802 | 0.0891 | 0.0135 | 4.61E-11 |
| rs3814883 | 16 | 29994922 | C | T | 0.542 | 0.071904 | 0.0111 | 7.82E-11 |
| rs4129585 | 8 | 143312933 | A | C | 0.456 | 0.078099 | 0.0109 | 7.70E-13 |
| rs4144795 | 2 | 233562731 | C | G | 0.375 | 0.0787 | 0.0113 | 3.12E-12 |
| rs4391122 | 5 | 60598543 | A | G | 0.49 | -0.0785 | 0.0109 | 5.70E-13 |
| rs4481150 | 3 | 52837793 | T | C | 0.542 | 0.069097 | 0.0109 | 2.29E-10 |
| rs4666990 | 2 | 185663304 | T | C | 0.559 | 0.074198 | 0.0109 | 1.14E-11 |
| rs4685 | 2 | 198257795 | T | C | 0.341 | 0.075896 | 0.0115 | 4.56E-11 |
| rs4702 | 15 | 91426560 | G | A | 0.458 | 0.078502 | 0.0114 | 4.90E-12 |
| rs4766428 | 12 | 110723245 | C | T | 0.541 | -0.0656 | 0.011 | 2.76E-09 |
| rs4801131 | 18 | 52752700 | C | T | 0.605 | 0.068204 | 0.011 | 6.01E-10 |
| rs55661361 | 11 | 124613957 | G | A | 0.679 | 0.077702 | 0.0116 | 1.89E-11 |
| rs5757730 | 22 | 39967430 | A | G | 0.464 | -0.073 | 0.0114 | 1.56E-10 |
| rs58120505 | 7 | 2029867 | T | C | 0.605 | 0.082704 | 0.0111 | 7.33E-14 |
| rs6065094 | 20 | 37453194 | A | G | 0.307 | -0.0751 | 0.0116 | 1.11E-10 |
| rs61937595 | 12 | 57682956 | C | T | 0.9173 | 0.144402 | 0.0206 | 2.37E-12 |
| rs6456771 | 6 | 27232395 | G | A | 0.717 | 0.077897 | 0.0119 | 6.47E-11 |
| rs6694545 | 1 | 30437268 | A | G | 0.25 | 0.0787 | 0.0127 | 6.06E-10 |
| rs707916 | 6 | 31697558 | G | A | 0.66 | 0.0887 | 0.0114 | 7.02E-15 |
| rs7085104 | 10 | 104628873 | A | G | 0.666 | 0.097399 | 0.0114 | 1.37E-17 |
| rs715299 | 6 | 32189841 | T | G | 0.703 | 0.079403 | 0.0119 | 2.72E-11 |
| rs72934570 | 18 | 53533189 | C | T | 0.9295 | 0.144795 | 0.0207 | 2.68E-12 |
| rs72986630 | 19 | 11849736 | C | T | 0.9299 | -0.1446 | 0.0239 | 1.47E-09 |
| rs73191547 | 8 | 10033425 | A | T | 0.659 | -0.06731 | 0.0115 | 4.58E-09 |
| rs7893279 | 10 | 18745105 | T | G | 0.897 | 0.113802 | 0.0175 | 7.33E-11 |
| rs7951870 | 11 | 46373311 | T | C | 0.821 | -0.09199 | 0.0144 | 1.58E-10 |
| rs832187 | 3 | 63833050 | C | T | 0.387 | 0.068996 | 0.0112 | 7.33E-10 |
| rs9263985 | 6 | 31190538 | G | A | 0.633 | -0.0732 | 0.0113 | 1.08E-10 |
| rs9269458 | 6 | 32542148 | A | G | 0.333 | 0.076702 | 0.0125 | 8.79E-10 |
| rs9276931 | 6 | 32928984 | A | G | 0.917 | 0.168501 | 0.0194 | 3.18E-18 |
| rs9461856 | 6 | 33395199 | G | A | 0.471 | -0.0755 | 0.0109 | 3.82E-12 |
| rs9607782 | 22 | 41587556 | T | A | 0.734 | -0.0901 | 0.0129 | 2.60E-12 |
| rs9636107 | 18 | 53200117 | A | G | 0.508 | -0.0798 | 0.0108 | 1.86E-13 |
| rs9876421 | 3 | 36848316 | C | T | 0.644 | -0.078 | 0.0114 | 9.41E-12 |

| Table S4. 25 independent significant SNPs identified by the CPASSOC method | | | | | | | | | |  |  |  |  |
| --- | --- | --- | --- | --- | --- | --- | --- | --- | --- | --- | --- | --- | --- |
| SNP | CHR | BP | A1 | A2 | P_CPASSOC_ | Z_SCZeur_ | P_SCZeur_ | Z_SCZeas_ | P_SCZeas_ | Z_T2Deur_ | P_T2Deur_ | Z_T2Deas_ | P_T2Deas_ |
| rs10750397 | 11 | 128234144 | A | G | 7.44E-09 | -0.19 | 0.85 | 1.90 | 0.057 | 4.64 | 9.80E-06 | 4.43 | 9.57E-06 |
| rs10883846 | 10 | 104958244 | C | T | 1.71E-09 | -5.71 | 1.06E-08 | -3.077 | 0.0021 | -1.18 | 2.00E-01 | -1.82 | 0.069 |
| rs1127215 | 1 | 117532790 | C | T | 2.47E-08 | 1.70 | 0.0905 | 1.81 | 0.071 | 4.80 | 3.70E-06 | 3.35 | 0.00081 |
| rs1169288 | 12 | 121416650 | A | C | 1.99E-08 | -2.50 | 0.013 | -2.66 | 0.0079 | -4.18 | 3.70E-05 | -4.51 | 6.34E-06 |
| rs12449758 | 17 | 3884026 | A | G | 5.36E-09 | -2.08 | 0.037 | 0.68 | 0.5009 | -4.82 | 1.70E-06 | -3.96 | 7.65E-05 |
| rs13012438 | 2 | 60584283 | C | A | 1.01E-10 | -0.46 | 0.65 | 0.54 | 0.59 | 5.27 | 6.20E-08 | 4.72 | 2.38E-06 |
| rs1408579 | 10 | 101912194 | C | T | 6.73E-11 | 3.31 | 0.00093 | -1.52 | 0.13 | 5.20 | 4.70E-07 | 4.27 | 1.93E-05 |
| rs2621416 | 6 | 32741868 | T | C | 7.42E-10 | -2.42 | 0.016 | 0.24 | 0.81 | -5.00 | 3.40E-07 | -4.26 | 2.04E-05 |
| rs2648731 | 5 | 52072194 | G | A | 6.28E-09 | -0.51 | 0.61 | -1.26 | 0.21 | -4.83 | 3.00E-06 | -4.11 | 3.94E-05 |
| rs35929648 | 17 | 47002543 | A | G | 2.13E-09 | -3.66 | 0.00026 | -0.67 | 0.50 | -4.64 | 4.50E-06 | -4.40 | 1.06E-05 |
| rs4371763 | 5 | 45983222 | A | G | 1.22E-09 | -4.22 | 2.47E-05 | -5.2 | 2.04E-07 | -3.50 | 8.60E-04 | -1.85 | 0.064 |
| rs4809556 | 20 | 61271339 | G | A | 1.71E-09 | 3.24 | 0.0012 | 1.62 | 0.1056 | 4.90 | 2.70E-06 | 3.85 | 0.00012 |
| rs55652053 | 5 | 102337170 | T | A | 1.15E-08 | -1.35 | 0.18 | -1.017 | 0.31 | -5.00 | 7.30E-07 | -3.39 | 0.00070 |
| rs57230522 | 2 | 65660181 | A | G | 6.74E-09 | 1.91 | 0.056 | 1.40 | 0.16 | 4.33 | 2.80E-05 | 4.89 | 1.01E-06 |
| rs61921611 | 12 | 66367726 | T | C | 4.67E-09 | -4.52 | 6.14E-06 | 0.28 | 0.79 | -5.27 | 2.60E-07 | -2.55 | 0.011 |
| rs6798814 | 3 | 187683184 | A | G | 7.40E-10 | 1.13 | 0.26 | 0.84 | 0.4 | 5.00 | 1.70E-06 | 4.46 | 8.26E-06 |
| rs684214 | 17 | 40696915 | C | T | 2.76E-11 | 1.05 | 0.29 | -1.55 | 0.12 | -5.64 | 6.10E-08 | -4.08 | 4.50E-05 |
| rs73054305 | 19 | 46157046 | C | T | 3.06E-11 | -1.25 | 0.21 | -1.25 | 0.21 | -5.31 | 1.90E-07 | -4.77 | 1.83E-06 |
| rs751858 | 19 | 19602821 | G | C | 2.58E-08 | -3.17 | 0.0015 | -0.58 | 0.57 | -4.15 | 6.10E-05 | -4.64 | 3.41E-06 |
| rs7926389 | 11 | 46711854 | A | G | 1.64E-11 | -5.34 | 8.87E-08 | -4.70 | 2.73E-06 | -1.07 | 2.80E-01 | -2.36 | 0.018 |
| rs8179252 | 2 | 27746832 | A | C | 2.32E-08 | -3.57 | 0.00036 | -0.06 | 0.95 | -4.50 | 1.80E-05 | -3.88 | 0.00010 |
| rs9263789 | 6 | 31128329 | G | A | 3.38E-08 | -3.75 | 0.00019 | -3.02 | 0.0026 | -2.86 | 3.70E-03 | -4.99 | 6.15E-07 |
| rs9318681 | 13 | 80738877 | G | C | 2.86E-11 | 0.68 | 0. 5 | -1.16 | 0.25 | -5.27 | 1.40E-07 | -5.00 | 5.73E-07 |
| rs9324063 | 14 | 104006693 | G | T | 7.13E-10 | -5.38 | 6.87E-08 | -3.75 | 0.00018 | -2.55 | 1.30E-02 | -1.23 | 0.22 |
| rs9903269 | 17 | 37742383 | A | T | 2.69E-08 | 1.68 | 0.093 | 0.62 | 0.54 | 4.54 | 1.20E-05 | 4.07 | 4.69E-05 |

| Table S5. Effective genes (P Bonferroni <9.23E-6) identified in pituitary through using the Transcriptome-wide Cross-trait/ethnic meta-analysis | | | | | | | | | | | | | |  |
| --- | --- | --- | --- | --- | --- | --- | --- | --- | --- | --- | --- | --- | --- | --- |
| ID | PASSET | BetaASSET | Symbol | Location | Type(s) | BetaT2DEUR | PT2DEUR | BetaT2DEAS | PT2DEAS | BetaSCZEUR | PSCZEUR | BetaSCZEAS | PSCZEAS |  |
| ENSG00000236493.2 | 1.46E-37 | -2.522 | EIF2S2P3 | Other | other | -57.58 | 3.82E-28 | -5.15 | 5.31E-76 | -2.62 | 0.616725 | 0.26 | 0.356801323 |  |
| ENSG00000140382.14 | 1.81E-14 | 1.69 | HMG20A | Nucleus | transcription regulator | 2.53 | 8.61E-10 | 4.57 | 7.78E-22 | -0.26 | 0.521857 | 0.29 | 0.54172691 |  |
| ENSG00000140400.16 | 2.32E-13 | -2.367 | MAN2C1 | Cytoplasm | enzyme | -2.47 | 0.00011 | -4.33 | 3.56E-11 | -0.79 | 0.214101 | -1.95 | 0.002888279 |  |
| ENSG00000213221.4 | 2.85E-12 | 1.898 | DNLZ | Cytoplasm | other | 2.72 | 2.12E-06 | 4.52 | 2.42E-18 | 0.52 | 0.363051 | -0.28 | 0.595069797 |  |
| ENSG00000177971.8 | 2.78E-11 | 1.234 | IMP3 | Cytoplasm | other | 18.30 | 1.05E-05 | 1.50 | 1.08E-08 | 5.36 | 0.196479 | 0.88 | 0.000791343 |  |
| ENSG00000204410.14 | 2.85E-11 | 0.375 | MSH5 | Nucleus | enzyme | 0.53 | 0.000199 | 0.27 | 0.005166314 | 1.13 | 1.22E-15 | 0.06 | 0.555433835 |  |
| ENSG00000159199.13 | 1.92E-10 | 0.783 | ATP5MC1 | Cytoplasm | transporter | 1.20 | 3.87E-07 | 1.16 | 6.50E-06 | 0.64 | 0.00676 | 0.08 | 0.740705668 |  |
| ENSG00000148384.12 | 3.73E-10 | 1.582 | INPP5E | Cytoplasm | phosphatase | 1.62 | 0.001432 | 4.41 | 1.69E-18 | 0.29 | 0.564965 | -0.02 | 0.973170731 |  |
| ENSG00000214435.7 | 1.07E-09 | 2.111 | AS3MT | Cytoplasm | enzyme | -0.94 | 0.172683 | 0.52 | 0.453678467 | 5.85 | 1.79E-17 | 2.99 | 1.77E-05 |  |
| ENSG00000272501.1 | 1.18E-09 | 1.462 | XXbac-BPG299F13.17 | Other | other | 1.36 | 0.018934 | 2.06 | 9.01E-07 | 0.65 | 0.257932 | 1.34 | 0.001395537 |  |
| ENSG00000159210.9 | 1.58E-09 | 15.235 | SNF8 | Cytoplasm | enzyme | 21.49 | 1.00E-05 | 19.15 | 0.00026799 | 16.90 | 0.000515 | 2.09 | 0.690362676 |  |
| ENSG00000187664.8 | 2.39E-09 | 13.558 | HAPLN4 | Extracellular Space | other | 11.90 | 0.008151 | 17.40 | 0.000150083 | 23.00 | 3.16E-07 | 1.61 | 0.725760778 |  |
| ENSG00000256053.7 | 3.35E-09 | -1.524 | COA8 | Cytoplasm | other | -0.97 | 0.007936 | -13.50 | 0.061795194 | -2.06 | 1.61E-08 | 3.61 | 0.617114922 |  |
| ENSG00000197815.4 | 4.28E-09 | -1.242 | RP1_253P74 | Other | other | -1.24 | 0.003377 | -1.33 | 0.001599878 | -2.18 | 2.59E-07 | -0.22 | 0.608965779 |  |
| ENSG00000278765.1 | 5.75E-09 | -13.516 | RP5_890E165 | Other | other | -17.13 | 0.000164 | -13.63 | 0.004077481 | -12.30 | 0.006802 | -10.79 | 0.022959182 |  |
| ENSG00000137312.14 | 1.23E-08 | -23.671 | FLOT1 | Plasma Membrane | other | 5.46 | 0.353108 | NA | NA | -52.80 | 2.62E-19 | NA | NA |  |
| ENSG00000125741.4 | 1.48E-08 | -0.49 | OPA3 | Cytoplasm | other | -0.73 | 0.000744 | -0.88 | 2.51E-09 | 0.02 | 0.921808 | -0.22 | 0.132656388 |  |
| ENSG00000175662.17 | 1.59E-08 | -1.708 | TOM1L2 | Cytoplasm | transporter | -1.83 | 0.003226 | -1.59 | 0.006842565 | -3.19 | 3.05E-07 | -0.40 | 0.498670068 |  |
| ENSG00000204520.12 | 2.56E-08 | -1.409 | MICA | Plasma Membrane | other | -0.54 | 0.25854 | -3.04 | 1.29E-08 | -2.07 | 1.76E-05 | -0.03 | 0.953698201 |  |
| ENSG00000204469.12 | 3.07E-08 | -1.165 | PRRC2A | Cytoplasm | other | -1.14 | 0.000136 | 14.06 | 0.00054258 | -1.27 | 2.08E-05 | -1.94 | 0.63297577 |  |
| ENSG00000140391.14 | 5.85E-08 | 1.225 | TSPAN3 | Plasma Membrane | other | 16.20 | 0.002672 | 2.28 | 1.15E-12 | 1.08 | 0.841666 | 0.12 | 0.702781631 |  |
| ENSG00000272221.1 | 7.10E-08 | -1.311 | XXbac-BPG181B23.7 | Other | other | -1.56 | 0.000629 | -3.20 | 1.01E-09 | -0.70 | 0.125258 | 0.10 | 0.847984613 |  |
| ENSG00000248278.1 | 7.20E-08 | 18.053 | SUMO2P17 | Other | other | 25.42 | 0.000145 | 27.32 | 4.77E-05 | 20.70 | 0.001969 | -1.32 | 0.844734405 |  |
| ENSG00000002919.14 | 1.21E-07 | 0.594 | SNX11 | Cytoplasm | transporter | 0.88 | 0.000149 | 0.52 | 0.017796496 | 0.53 | 0.022149 | 0.48 | 0.028539586 |  |
| ENSG00000140995.16 | 1.34E-07 | -0.509 | DEF8 | Other | other | -0.40 | 0.003652 | -0.36 | 0.929707472 | -0.62 | 6.51E-06 | -5.16 | 0.204831262 |  |
| ENSG00000172922.8 | 1.67E-07 | -0.802 | RNASEH2C | Other | other | -1.00 | 0.002348 | -0.89 | 0.002121078 | -1.05 | 0.001378 | -0.37 | 0.19738726 |  |
| ENSG00000095485.16 | 2.09E-07 | -25.235 | CWF19L1 | Other | other | -49.24 | 3.40E-07 | -42.27 | 1.58E-05 | -23.70 | 0.014109 | 14.90 | 0.127991538 |  |
| ENSG00000127870.16 | 2.87E-07 | 10.461 | RNF6 | Nucleus | transcription regulator | 8.53 | 0.03125 | 25.99 | 6.23E-10 | 7.75 | 0.050311 | 0.15 | 0.972267032 |  |
| ENSG00000157823.16 | 3.48E-07 | 1.567 | AP3S2 | Cytoplasm | transporter | 3.41 | 1.99E-08 | 4.45 | 9.13E-13 | -0.88 | 0.146772 | -0.68 | 0.275771146 |  |
| ENSG00000163848.19 | 3.94E-07 | -0.874 | ZNF148 | Nucleus | transcription regulator | -0.99 | 0.00413 | -1.78 | 2.42E-07 | -0.16 | 0.646657 | -0.57 | 0.09654459 |  |
| ENSG00000075413.17 | 5.28E-07 | -1.341 | MARK3 | Cytoplasm | kinase | -2.27 | 2.87E-05 | -0.87 | 0.100392865 | -1.56 | 0.004011 | -0.73 | 0.165324185 |  |
| ENSG00000277072.4 | 6.10E-07 | 0.614 | STAG3L2/STAG3L3 | Other | other | 1.03 | 1.46E-05 | 0.29 | 0.253469301 | 0.65 | 0.006468 | 0.42 | 0.104131314 |  |
| ENSG00000262319.1 | 7.67E-07 | 12.306 | CTC_457L162 | Other | other | 7.84 | 0.123933 | 3.36 | 0.489675742 | 24.00 | 2.52E-06 | 14.66 | 0.002585251 |  |
| ENSG00000260778.5 | 7.93E-07 | -1.312 | MIR3677HG | Other | other | -2.30 | 0.703947 | -1.52 | 5.41E-05 | -9.07 | 0.134856 | -1.07 | 0.004483635 |  |
| ENSG00000272858.1 | 1.00E-06 | -0.639 | CTA_292E108 | Other | other | -0.36 | 0.223596 | -1.21 | 3.30E-07 | -0.94 | 0.001359 | -0.05 | 0.828961436 |  |
| ENSG00000253893.2 | 1.04E-06 | -1.342 | FAM85B | Other | other | -1.71 | 0.004054 | -0.83 | 0.104665746 | -2.87 | 1.51E-06 | -0.45 | 0.378166018 |  |
| ENSG00000175749.11 | 1.10E-06 | 1.071 | EIF3KP1 | Other | other | 1.99 | 5.80E-06 | 1.37 | 0.001879183 | 0.48 | 0.27497 | 0.44 | 0.313384314 |  |
| ENSG00000196275.13 | 1.61E-06 | 1.492 | GTF2IRD2/GTF2IRD2B | Nucleus | other | 2.32 | 0.000235 | 0.79 | 0.195810199 | 1.98 | 0.001693 | 0.94 | 0.123994608 |  |
| ENSG00000103351.12 | 1.72E-06 | 1.12 | CLUAP1 | Nucleus | other | 2.14 | 1.87E-05 | 2.42 | 4.31E-08 | 0.69 | 0.169705 | -0.64 | 0.148713 |  |
| ENSG00000059915.16 | 1.73E-06 | 0.456 | PSD | Cytoplasm | transporter | -0.55 | 0.010769 | -0.01 | 0.933120588 | 1.70 | 2.21E-15 | 0.77 | 9.32E-06 |  |
| ENSG00000137310.11 | 1.84E-06 | 1.835 | TCF19 | Nucleus | transcription regulator | 2.02 | 0.000215 | 37.25 | 1.06E-06 | 1.40 | 0.010247 | 15.67 | 0.040143463 |  |
| ENSG00000229759.1 | 2.53E-06 | -1.277 | MRPS18AP1 | Other | other | -1.01 | 0.081115 | -1.27 | 0.013398539 | -1.54 | 0.007799 | -1.29 | 0.01189952 |  |
| ENSG00000276073.1 | 2.65E-06 | -1.365 |  |  |  | -2.58 | 1.90E-05 | -1.57 | 0.005132881 | -1.73 | 0.004107 | 0.21 | 0.709567718 |  |
| ENSG00000255556.2 | 2.91E-06 | 1.176 | RP11_351I216 | Other | other | 0.80 | 0.02544 | 9.94 | 0.116079989 | 1.56 | 1.14E-05 | -9.71 | 0.12494822 |  |
| ENSG00000259362.2 | 3.00E-06 | 12.382 | LOC101929457 | Other | other | 17.40 | 0.000567 | 32.34 | 7.57E-09 | 0.57 | 0.910715 | 0.78 | 0.889052374 |  |
| ENSG00000184988.8 | 3.06E-06 | -0.888 | TMEM106A | Cytoplasm | other | 0.98 | 0.827289 | -1.26 | 3.19E-06 | -9.74 | 0.029588 | -0.50 | 0.066445005 |  |
| ENSG00000239510.2 | 3.28E-06 | 0.971 | RPL9P5 | Other | other | 1.15 | 0.008635 | 1.74 | 1.33E-05 | 0.26 | 0.550959 | 0.64 | 0.107150537 |  |
| ENSG00000188825.13 | 3.77E-06 | -0.707 | LINC00910 | Other | other | -7.96 | 0.12693 | -0.94 | 1.34E-05 | -4.95 | 0.342517 | -0.45 | 0.036833047 |  |
| ENSG00000249188.1 | 3.77E-06 | -0.734 | ENPP7P1 | Other | other | -0.77 | 0.019445 | -0.37 | 0.220553624 | -1.70 | 3.04E-07 | -0.24 | 0.427344562 |  |
| ENSG00000085788.13 | 4.51E-06 | -0.816 | DDHD2 | Cytoplasm | enzyme | -0.04 | 0.897595 | -0.02 | 0.970346975 | -1.24 | 3.68E-05 | -2.46 | 1.03E-07 |  |
| ENSG00000253426.5 | 4.94E-06 | 0.56 |  |  |  | 0.72 | 0.000213 | 0.01 | 0.986361065 | 0.63 | 0.001388 | 0.27 | 0.465543912 |  |
| ENSG00000263412.1 | 5.00E-06 | 0.465 | NFE2L1-DT | Other | other | 0.54 | 0.023749 | 0.41 | 0.021844807 | 0.60 | 0.012901 | 0.40 | 0.026630685 |  |
| ENSG00000237541.3 | 5.25E-06 | 1.467 | HLA-DQA2 | Plasma Membrane | transmembrane receptor | 4.63 | 3.53E-13 | 2.12 | 0.001172932 | -0.43 | 0.501312 | -0.51 | 0.432591209 |  |
| ENSG00000164048.13 | 5.68E-06 | -1.69 | ZNF589 | Nucleus | transcription regulator | -1.60 | 0.039609 | -1.39 | 0.052075123 | -1.87 | 0.015883 | -1.91 | 0.007681994 |  |
| ENSG00000100395.14 | 6.72E-06 | 0.468 | L3MBTL2 | Nucleus | transcription regulator | 0.52 | 0.000392 | 13.50 | 0.004121908 | 0.40 | 0.006328 | 0.73 | 0.876725831 |  |
| ENSG00000166965.12 | 7.55E-06 | 1.063 | RCCD1 | Other | other | 0.47 | 0.32037 | 5.90 | 1.32E-35 | -0.49 | 0.308147 | -1.66 | 0.000471413 |  |
| ENSG00000170100.13 | 7.63E-06 | -0.61 | ZNF778 | Nucleus | transcription regulator | -0.69 | 0.004592 | -0.51 | 0.10752568 | -0.66 | 0.007107 | -0.50 | 0.111045502 |  |
| ENSG00000178773.14 | 8.20E-06 | 0.67 | CPNE7 | Cytoplasm | transporter | 0.45 | 0.036157 | 8.27 | 0.127122273 | 0.89 | 2.79E-05 | -4.56 | 0.400250903 |  |
| ENSG00000164615.4 | 8.31E-06 | 0.995 | CAMLG | Cytoplasm | other | 1.16 | 0.011259 | 1.65 | 0.000154616 | 0.69 | 0.13366 | 0.47 | 0.2805079 |  |
| ENSG00000197181.11 | 8.39E-06 | 1.4 | PIWIL2 | Cytoplasm | enzyme | 1.30 | 0.029402 | 2.09 | 0.001664215 | 1.45 | 0.01525 | 0.77 | 0.247054914 |  |
| ENSG00000280273.2 | 8.97E-06 | -0.423 | AF1312161 | Other | other | -0.46 | 0.000669 | -5.35 | 0.228732404 | -0.39 | 0.003835 | 6.90 | 0.120891205 |  |
